# Evaluation of anticancer activity in vitro of a stable copper(I) complex with phosphine-peptide conjugate

**DOI:** 10.1038/s41598-021-03352-2

**Published:** 2021-12-14

**Authors:** Urszula K. Komarnicka, Barbara Pucelik, Daria Wojtala, Monika K. Lesiów, Grażyna Stochel, Agnieszka Kyzioł

**Affiliations:** 1 Faculty of Chemistry, University of Wroclaw, Joliot-Curie 14, 50-383 Wroclaw, Poland; 2 Małopolska Center of Biotechnology, Jagiellonian University, Gronostajowa 7A, 30-387 Kraków, Poland; 3 Faculty of Chemistry, Jagiellonian University, Gronostajowa 2, 30-387 Kraków, Poland

**Keywords:** Chemical biology, Inorganic chemistry

## Abstract

[CuI(2,9-dimethyl-1,10-phenanthroline)P(p-OCH_3_-Ph)_2_CH_2_SarcosineGlycine] (1-MPSG), highly stable in physiological media phosphino copper(I) complex—is proposed herein as a viable alternative to anticancer platinum-based drugs. It is noteworthy that, 1-MPSG significantly and selectively reduced cell viability in a 3D spheroidal model of human lung adenocarcinoma (A549), in comparison with non-cancerous HaCaT cells. Confocal microscopy and an ICP-MS analysis showed that 1-MPSG effectively accumulates inside A549 cells with colocalization in mitochondria and nuclei. A precise cytometric analysis revealed a predominance of apoptosis over the other types of cell death. In the case of HaCaT cells, the overall cytotoxicity was significantly lower, indicating the selective activity of 1-MPSG towards cancer cells. Apoptosis also manifested itself in a decrease in mitochondrial membrane potential along with the activation of caspases-3/9. Moreover, the caspase inhibitor (Z-VAD-FMK) pretreatment led to decreased level of apoptosis (more pronouncedly in A549 cells than in non-cancerous HaCaT cells) and further validated the caspases dependence in 1-MPSG-induced apoptosis. Furthermore, the 1-MPSG complex presumably induces the changes in the cell cycle leading to G2/M phase arrest in a dose-dependent manner. It was also observed that the 1-MPSG mediated intracellular ROS alterations in A549 and HaCaT cells. These results, proved by fluorescence spectroscopy, and flow cytometry, suggest that investigated Cu(I) compound may trigger apoptosis also through ROS generation.

Currently cancer is a leading cause of death worldwide. Due to lack of drug selectivity, the administration of most drugs in cancer therapies is associated with severe side effects. Another major problem in current chemotherapies is the rapid drug resistance acquisition by tumor cells. These are some of the most important reasons why completely new anticancer drugs are desperately needed^1^. In recent years there has been a growing interest in developing metal-based complexes for cancer treatment. Metals show unique characteristics, such as redox activity, variable coordination modes, reactivity towards organic substrates and, importantly, they exhibit a promising in vitro cytotoxic activity against different cancer cells^2,3^ Platinum drugs (cisplatin and the second and third-generation analogues such as carboplatin and oxaliplatin) are among the metal-based complexes most commonly used in anticancer treatment.

Regrettably, the clinical use of cisplatin and other platinum drugs is hampered by their high systemic toxicity and drug resistance. Moreover, the mode of action of DNA targeting agents, such as platinum drugs, is associated with DNA damage postponing DNA replication, which results in cell death. If not repaired properly, many of these genomic changes can cause gene mutations or chromosomal alterations. In fact, approximately 10 years after chemotherapy with cisplatin some patients may develop cancer due to cisplatin-induced DNA lesions^4–6^. Thus, it is highly desirable that anticancer drugs selectively kill only rapidly dividing cells while keeping intact healthy cells^5,6^.

At present, the design and development of anticancer therapeutics targeting cellular mitochondria and omitting DNA attract increasing attention^7–9^. It is noteworthy that the mitochondria in tumor cells significantly differ from those in normal cells^10–12^. The differences mainly stem from different pathways of the highly efficient glucose metabolism (even irrespective of oxygen availability) in cancer cells^13^. Moreover, mitochondria play a crucial role not only in tumorigenesis, but also in tumor progression. Therefore they are proposed as the main targets of novel selectively acting chemotherapeutics. Thus, there are many advantages of mitochondria as molecular targets^14^: (*i*) as mitochondria are the powerhouse of the eukaryotic cell, a disruption of the mitochondrial function stops the rapid growth of cancer cells; (*ii*) the mitochondrial release of cytochrome c results in the activation of the caspase-dependent apoptotic pathway; (*iii*) the nucleotide excision repair (NER) pathway, reducing the efficacy of any nuclear DNA targeting anticancer drug, is avoided; and (*iv*) plausible perturbations in many important cancer cell metabolism pathways.

The aforementioned constraints have led to an intensive search for new metal-based agents exhibiting better selectivity, lower toxicity and improved anticancer activity based on alternative targets and mechanisms of bioactivity^15–17^. Although the chemistry of copper has been dominated essentially by copper(II) compounds, mainly due to the difficulty in stabilizing copper(I) species, some examples of copper(I) complexes (mostly involving *P* donor and *N, N*-diimine systems) showing considerable cytotoxic activity been reported^18–23^. Importantly, owing to the soft nature of the *P*-donor atoms, phosphine ligands can efficiently stabilize Cu(I)-D10 metal centers and the latter can be easily functionalized. For instance, the conjugation of peptides via the phosphine moiety to copper(I) complexes can enable selective delivery to cancer cells. In addition, peptide carriers enable specific interactions with the receptors over-expressed on tumor cells^24–27^. It was reported that the tripeptide Arg-Gly-Asp motif (RGD) linked with anticancer drugs, such as paclitaxel, fluorouracil and doxorubicin, significantly increased cytotoxicity and decreased toxicity against healthy cells in comparison with native drugs^28–33^. Also, a small SarGly dipeptide was used as a positron emission tomography (PET) tracer targeted to the H^+^/peptide transporters (PEPTs), functionally expressed in some human cancer cell lines, for cancer detection in mice^34,35^.

Our recently published results on the bioactivity of new Cu(I) complexes, including the complex with phosphine P(*p-*OCH_3_-Ph)_2_CH_2_OH and P(*p-*OCH_3_-Ph)_2_CH_2_SarGly (**1-MPSG**)^36^ encouraged us to continue our research using a more advanced 3D in vitro cell culture model (*i.e.*, spheroids). 3D tumor spheroids have several advantages which can overcome the limitations of conventional 2D cultures, including the ability to mimic the complexity and heterogeneity of tumors as well as their architecture, which monolayer cell cultures are unable to reproduce^37^. Unlike adherent cultures, spheroids can provide a microenvironment which closely mimics the cellular interactions observed in tumor tissues^38,39^. This paper is a continuation of our previous study on the structure–reactivity relationships of copper(I) complexes bearing phosphine ligands and on the evaluation of their cytotoxic activity in vitro^29–34^. We have recently reported the synthesis of phosphine ligands with and without sarcosine-glycine (**SarGly)** peptide (P(*p-*OCH_3_*-*Ph)_2_CH_2_OH (**MPOH**) and P(*p-*OCH_3_*-*Ph)_2_CH_2_**SarGly** (**MPSG**), respectively, and the resulting two copper(I) complexes ([CuI(2,9-dimethyl-1,10-phenanthroline)**MPOH**] (**1-MPOH)** and [CuI(2,9-dimethyl-1,10-phenanthroline)**MPSG**] (**1-MPSG**); Fig. S1, Supporting Information). The cytotoxicity of the compounds was evaluated in vitro against colon, lung, breast, pancreatic and prostate tumor cell lines and non-tumor lung, kidney and keratinocyte cell lines. The introduction of the peptide motif significantly increased the selectivity towards cancer cells. **1-MPSG** showed its unusually low genotoxicity and was able to generate reactive oxygen species owing to redox processes^36^. On the basis of the findings mentioned above, in this study we carried out a preclinical investigation into the therapeutic potential of **1-MPSG** in 3D lung cancer cell cultures and proposed an explanation of the working mechanism of this new copper(I) complex.

## Results and discussion

### Cellular uptake of Cu(I) complexes

**Figure 1:**
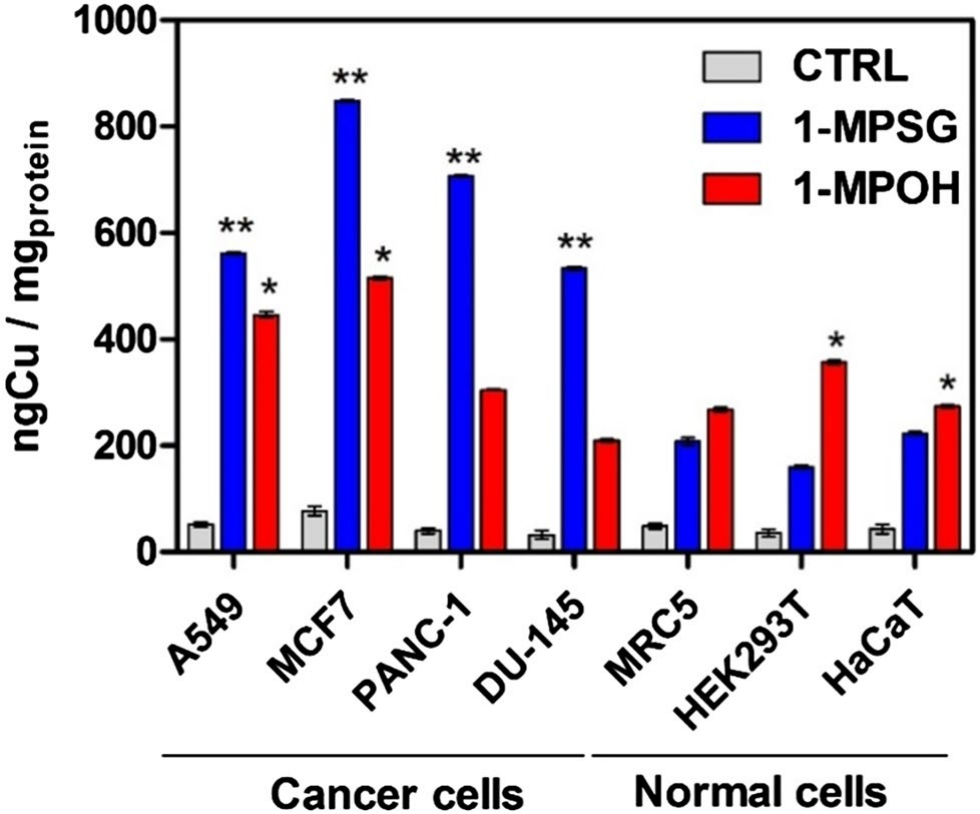
Final intracellular copper concentration expressed by ng Cu/mg protein for complexes **1-MPOH** and **1-MPSG** in c = 1 µM: after 24 h of incubation with cancer or normal lines. Data are expressed as mean ± SEM. The asterisks denote *p*-values < *0.05, **0.01 compared to control (Two-way ANOVA followed by Bonferroni multiple comparisons test was performed using GraphPad Prism version 5.0.0 for Windows, GraphPad Software, San Diego, California USA, www.graphpad.com.).

An ICP-MS analysis was performed to correlate the cytotoxicity determined in vitro by us in our previous study^36^ with the cellular uptake of the investigated complexes and to evaluate the role of peptide carrier **HSG** in the selective delivery of the copper(I) compounds. The intracellular copper accumulation in A549, MCF7, PANC-1, MRC5, HEK293T and HaCaT cells treated for 24 h with **1-MPOH** and **1-MPSG** was assessed (Fig. 1).

In the case of all the selected tumor cell lines (A549, MCF-7, PANC-1, DU-145) after incubation with **1-MPSG**, a significant increase in copper accumulation was detected in comparison with the control normal cells (MRC5, HEK293T, HaCaT)). This means that the copper(I) compound had been effectively internalized by the studied cancer cells (A549: 63%, MCF7: 75%, PANC-1: 69%, DU-145: 61%). **1-MPOH** (the copper(I) complex without the peptide motif) is accumulated inside cancer cells significantly less efficiently (less than 50% of its initial concentration is accumulated) at the same conditions. This phenomenon can be directly connected with the cytotoxicity of the investigated complex^36^ and can be explained by the excellent transporting potential of the dipeptide (SarGly). Thus, the introduction of the peptide motif into the structure of the investigated metal complex resulted in an increased concentration of the copper(I) compound inside the cancer cells and consequently, in augmented cytotoxic activity in vitro.

Notably, the accumulation of copper significantly differed between the cancer lines and the normal lines. In the case of the non-cancerous cell lines (MCR5, HEK293T, HaCaT), copper accumulation for the **1-MPSG** complex amounted to only *c.a.* 20%. These results fully corroborate the differences between the cytotoxicity of **1-MPSG** against the cancer lines (IC_50_ = A549: 1.00 ± 2.2 µM; MCF7: 0.63 ± 0.08 µM; PANC-1: 1.76 ± 1.2 µM; DU-145: 5.65 ± 7.2 µM)^36^ and against the normal cell lines (IC_50_ = MRC5: 80.54 ± 2.7 µM; HEK293T: 77.34 ± 4.1 µM; HaCaT: 71.76 ± 3.2 µM)^36^. To conclude, it is suggested that the high selectivity and cytotoxicity in vitro of **1-MPSG** can be directly connected with the presence of the peptide motif (**SarGly**) and its role as a targeting ligand. In the current literature, ^11^C-glycylsarcosine (^11^C-Gly-Sar) is described as a highly selective motif for the PEPTs (H+/peptide transporters) expressed on human cancer cells^40–42^.

**Figure 2:**
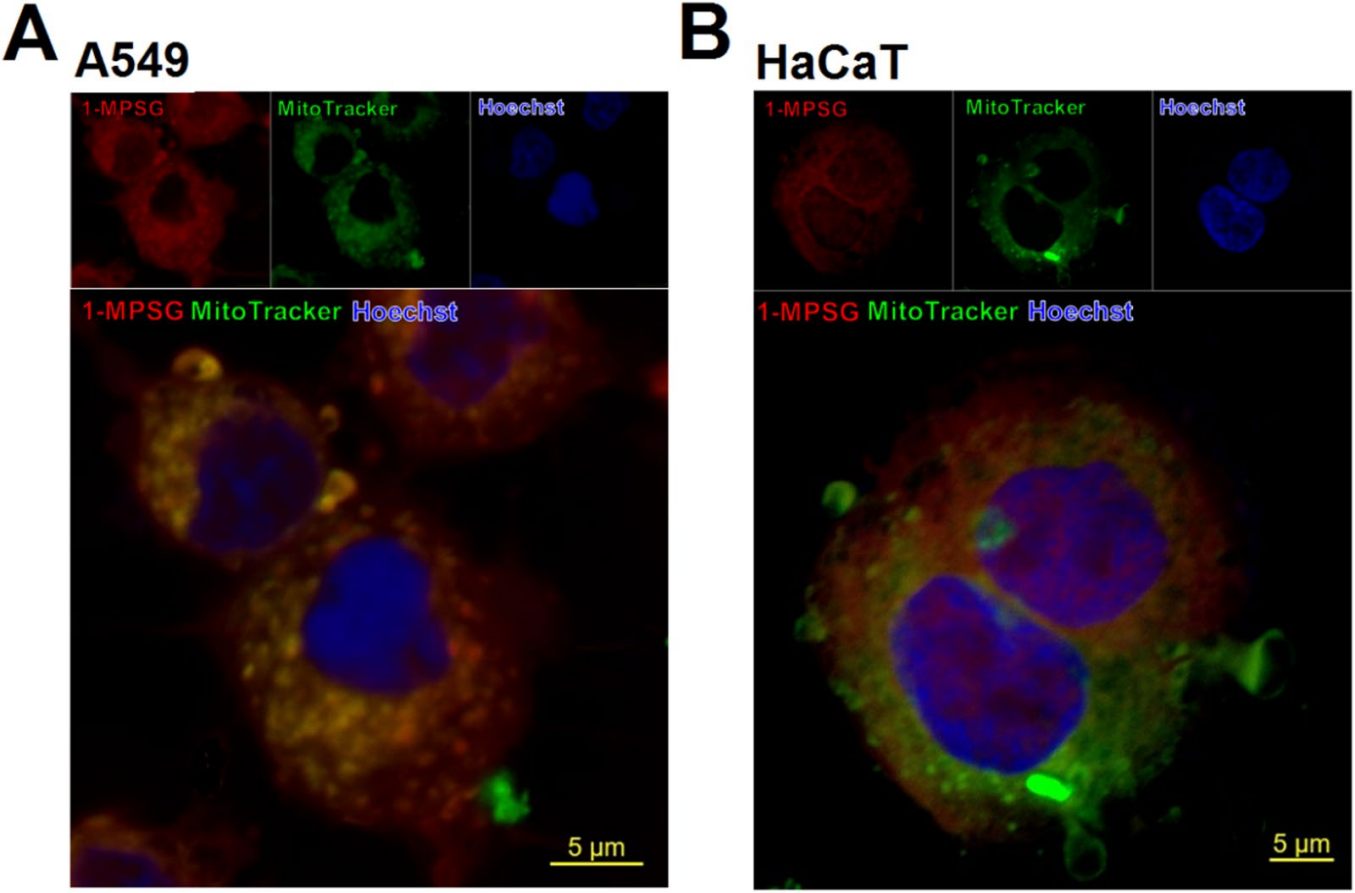
Colocalization images of **1-MPSG** with MitoTracker Green in (**A**) A549 cells and (**B**) HaCaT cells. The cells were treated with 1 µM **1-MPSG** for 2 h and then co-incubated with 200 nM MitoTracker Green for 1 h and counterstained with Hoechst 33,342.

In order to explore the subcellular localization of **1-MPSG**, a commercially available nucleus staining probe (Hoechst 33342) and a mitochondria-labeling probe (MitoTracker) were used for colocalization analyses (Fig. 2, S2 and S3, Supporting Information). The **1-MPSG** colocalization analyses with Hoechst 33342 and MitoTracker consisted in quantifying Pearson’s correlation coefficient. The Pearson correlation coefficients (R) of the green and red fluorescent signals were relatively higher for the A549 cells than the HaCaT cells (0.89 and 0.40, respectively). A similar analysis carried out for **1-MPSG** and Hoechst colocalization also showed a higher Pearson correlation coefficient for the cancer cells than the non-cancerous cells: 0.52 and 0.38, respectively (Figs. S2–S4, Supporting Information). These results thus confirmed both the mitochondrial and nuclear localization of **1-MPSG**. Furthermore**, the 1-MPSG** compound can be effectively taken up by A549 cells and accumulated in other organelles, as demonstrated in Figs. S2–S8 in Supporting Information. Confocal imaging showed that the **1-MPSG** fluorescence was overlapped with that generated by LysoTracker (lysosomes staining). **1-MPSG** fluorescence was additionally observed in the whole cytoplasm, whereas only a little colocalization was detected for ERTracker (endoplasmic reticulum). Therefore, it can be supposed that **1-MPSG** induced cell death probably through the mitochondrial pathway, but DNA may also be the target. Moreover, the cytoplasmic localization indicates that in contrast to platinum-anticancer drugs, these complexes may exert their anticancer activity through interaction with proteins in the cytoplasm. Single stained controls of Hoechst 33342, MitoTracker Green, and **1-MPSG** as well as colocalization images of **1-MPSG** with MitoTracker Green and counterstained with Hoechst 33342 are presented in Figs. S7 and S8 (Supporting Information).

Based on these studies it can be concluded that the synthesized Cu(I) complex is able to localize in both nuclei and mitochondria, and that the mechanism of its anticancer action is multi-modal. We hypothesize that targeting only one organelle may not be able to reach the expected therapeutic effect. One possible mode of action exhibited by **1-MPSG** is to simultaneously target multiple subcellular organelles or structures. Based on the confocal imaging of **1-MPSG** localization we indicated that **1-MPSG** could accumulate in the selected important targeted—nucleus and mitochondria of cancer cells to maximize the expected biological effect. It should be noticed that the vast majority of conventional chemotherapeutic drugs need to work in the nucleus of cancer cells to induce apoptosis^43–45^. On the other hand, mitochondria are also important as targets of anticancer drugs. Many anticancer drugs act on mitochondria and activate the apoptotic cell death^46^. Similar to other, well-known anticancer drugs (*e.g.* Paclitaxel or doxorubicin)^47–50^ which, in addition to acting on recognized targets, also act on mitochondria to varying degrees to induce apoptosis. Thus, we also indicate that one (but not only) of the targets of **1-MPSG** action can be mitochondria.

The obtained data are in agreement with our previous reports on DNA interactions with **1-MPOH** and **1-MPSG**^36^. Fluorescence spectroscopic data (CT-DNA titration), together with an analysis of DNA fragmentation (gel electrophoresis) and molecular docking, provided evidence for the multimodal interaction with DNA with a predominance of groove binding, presumably related to negligible genotoxicity^36^. Knowing that many first-line anticancer agents (*e.g.*, doxorubicin, camptothecin, cisplatin, and Pt-based drugs) interfere with DNA or its associated enzymes, we can postulate a similar mode of action. Thus, the above-mentioned compounds along with our novel metal complexes can exhibit the activity associated with one or more of the following actions: (*i*) interaction with nuclear DNA by intercalation leading to the inhibition of macromolecular biosynthesis, (*ii*) binding to DNA (*e.g.*, crosslinking) or (*iii*) binding to and/or stabilization of topoisomerase I and the formed DNA complexes. All these, in consequence, lead to cancer cell death^51,52^.

### Determination of apoptotic cell death

**Figure 3:**
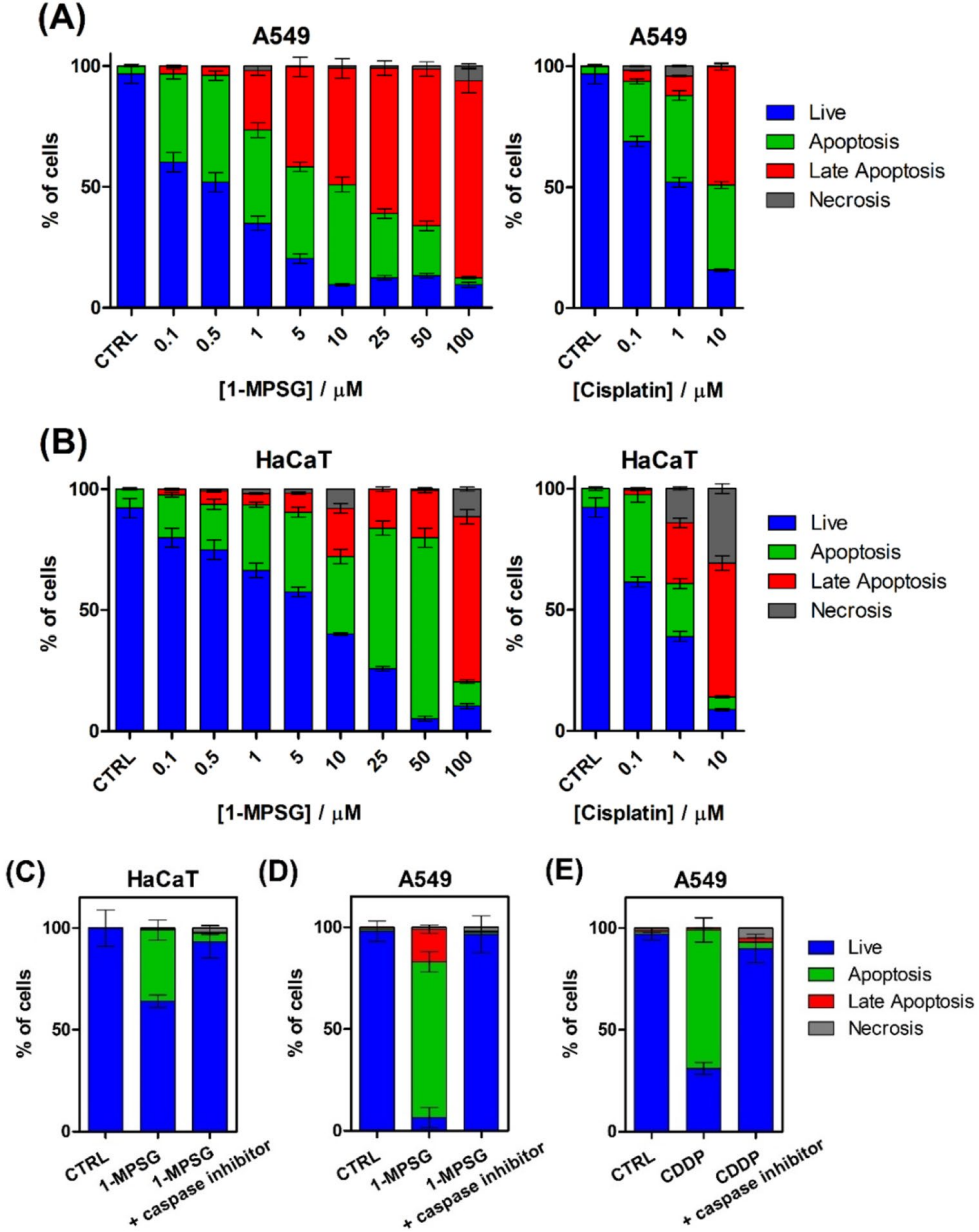
Determination of (**A**) A549 and (**B**) HaCaT cell death modes induced by 1-MPSG: the Annexin V-FITC/propidium iodide (PI) double staining assay (Annexin V-FITC—green fluorescence, PI—red fluorescence) was used to detect phosphatidylserine externalization in apoptosis and analyze the membrane integrity, respectively. FACS quantified apoptosis and necrosis after Annexin V-FITC and PI labelling. The effect of caspase inhibitor (Z-VAD-FMK) on the (**C**) HaCaT cells treated with 1 μM 1-MPSG, (**D**) A549 cells treated with 1 μM 1-MPSG and (**E**) A549 cells treated with 1 μM CDDP. Data are expressed as mean ± SEM. (The figure was plotted using GraphPad Prism version 5.0.0 for Windows, GraphPad Software, San Diego, California USA, www.graphpad.com.).

Considering that many anticancer drugs exert their antitumor effects by apoptosis activation^53^, the features related to this death pathway were investigated. Apoptosis is a highly privileged cell death mode in discovering novel antitumor agents, mainly because of its self-regulated and well-programmed strategy to maintain homeostasis^36,54,55^. Flow cytometry was applied to quantitatively determine the type of cell death induced by the **1-MPSG** compound. Figure 3 reports the results of a representative experiment as percentages of apoptotic cells (Annexin V-FITC-positive and PI-negative), late apoptotic cells (Annexin V-FITC positive and PI-positive) and necrotic cells (Annexin V-FITC negative and PI-positive) measured 24 h after treatment with **1-MPSG** at different concentrations, while Figs. S9–S11 (Supporting Information) show the dot plots. In the experiment, cisplatin was used as a reference compound.

The obtained data indicated that **1-MPSG** shows relative selectivity towards cancer cells, especially at lower concentrations (up to 5 μM). For instance, in the A549 cells treated with **1-MPSG,** apoptotic cells were found to amount to 36% and live cells to 60% (Fig. 3A) while in the case of HaCaT cells, apoptotic cells amounted to 18% and viable cells to 75% (Fig. 3B). An analysis of the percentage of live, apoptotic and necrotic cells upon treatment with the **1-MPSG** complex revealed concentration-dependent apoptosis in the A549 cells and lower cytotoxicity in the HaCaT cells. In contrast, necrotic death was marginal (less than 1%).

In comparison with cisplatin, **1-MPSG** leads to a similar level of apoptosis in A549 cells and a lower level of apoptosis in non-cancerous HaCaT cells (cisplatin induces cell death in the same manner in both the tested cancer and non-cancerous cell lines) (Fig. 3). Moreover, cisplatin may induce necrosis in HaCaT cells after treatment with a higher concentration (30% necrotic cells after the use of 10 μM), whereas **1-MPSG** leads to, more preferably, late apoptosis. In the case of the HaCaT cells (the non-cancerous reference cell line), almost a double sparing effect was observed. This clearly indicates a huge potential of the **1-MPSG** complex for selective action and the reduction of systemic toxicity. Moreover, in comparison with the reference drug (cisplatin) a significant reduction in doses causing a toxic effect (the percentage of apoptotic and necrotic cells) is evident in the case of both the tested cell lines.

Additionally, a flow cytometry analysis was performed to measure the caspase-dependent apoptosis triggered by **1-MPSG** treatment in both the A549 and HaCaT cells. As a reference, we used CDDP as a potent apoptosis inducer^56^. Z-VAD-FMK (a caspase inhibitor) was used^41,42^ to validate the caspase-dependent mitochondrial pathway of apoptosis induced by **1-MPSG**. The cells were pre-treated for 2 h with 20 µM Z-VAD-FMK and then incubated with 1 μM **1-MPSG** or 1 μM CDDP for 24 h (Figs. 3C–E, S11, Supporting Information). The obtained data show that pre-incubation with the Z-VAD-FMK caspase inhibitor prevented an increase in apoptotic cells caused by **1-MPSG**. In the Z-VAD-FMK pre-treated cells, apoptosis induced by **1-MPSG** was almost completely inhibited in A549 cells as well as in HaCaT cells, but in the latter case the effect was much less pronounced.

### Mitochondrial damage and caspases activity

It is well known that, mitochondria play crucial role in apoptotic cell death. In brief, mitochondrial outer membrane permeabilization (MOMP) starts a signaling cascade leading to cancer cell death. Even though, the apoptosis is a major form of regulated cell death however it is not the only one death type (*e.g.*, necroptosis, pyroptosis and ferroptosis). It’s worth to mention that MOMP also has other significant consequences, *e.g.*, the induction of pro-inflammatory signaling^57^.

Taking into account the above results (Figs. 2, S2 and S3, Supporting Information), apoptotic cell death with the dysregulation of mitochondrial membrane potential (MMP) seems to be the most probable mode of action of **1-MPSG**. For a precise determination of the A549 and HaCaT cells death type caused by **1-MPSG** and a better explanation of the mechanism of this process the following experiments were carried out: (***i***) the measurement of the MMP level decreasing during apoptosis, (***ii***) the examination of the proteolytic activity of caspases 9 and 3, and (***iii***) the production of pro-inflammatory cytokines (IL-6 and TFN-α).

**Figure 4:**
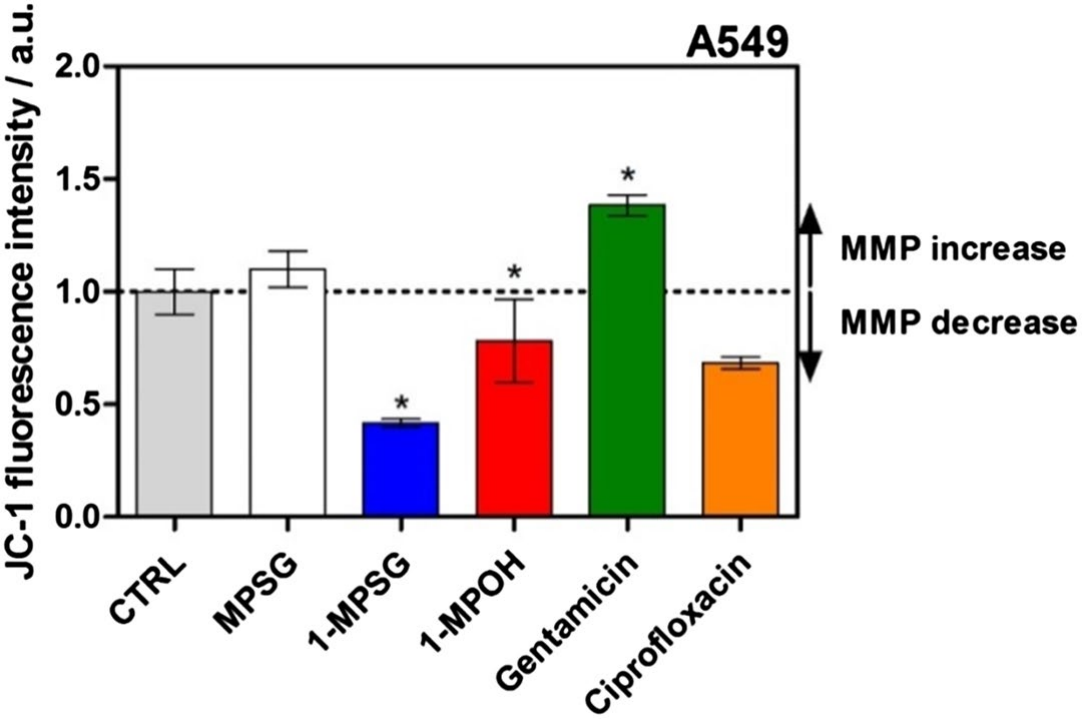
Influence of **1-MPOH** and **1-MPSG** complexes (c = 1 µM) on the intensity of JC-10 fluorescence in treated A549 cells. Alteration in MMP is given as an emission ratio 570 nm/530 nm. (Control (CTRL)—untreated cells, ciprofloxacin—a negative control, gentamicin—a positive control); *P < 0.05 compared to control). (Two-way ANOVA followed by Bonferroni multiple comparisons test was performed using GraphPad Prism version 5.0.0 for Windows, GraphPad Software, San Diego, California USA, www.graphpad.com.).

As it is clearly seen in Fig. 4 as well as Figs. S12 and S13 in Supporting Information, the **1-MPSG** complex significantly decreased MMP. The level of MMP after the treatment of the cells with **1-MPSG** was much lower than after the treatment with ciprofloxacin. This means that the complex caused permeability of the mitochondrial membrane. The cytochrome c can be released into the cytoplasm as a consequence of MMP decrease and then in the presence of ATP, interacts with the Apaf-1 factor and procaspase 9. This process usually starts a cascade of executive caspases, particularly caspase 3 (responsible for apoptotic cell death)^41,42^. Controls for the experiment indicating the influence of **1-MPSG** complexes on the intensity of JC-10 fluorescence in treated A549 and HaCaT cells are presented in Fig. S13 (Supplementary Materials).

**Figure 5:**
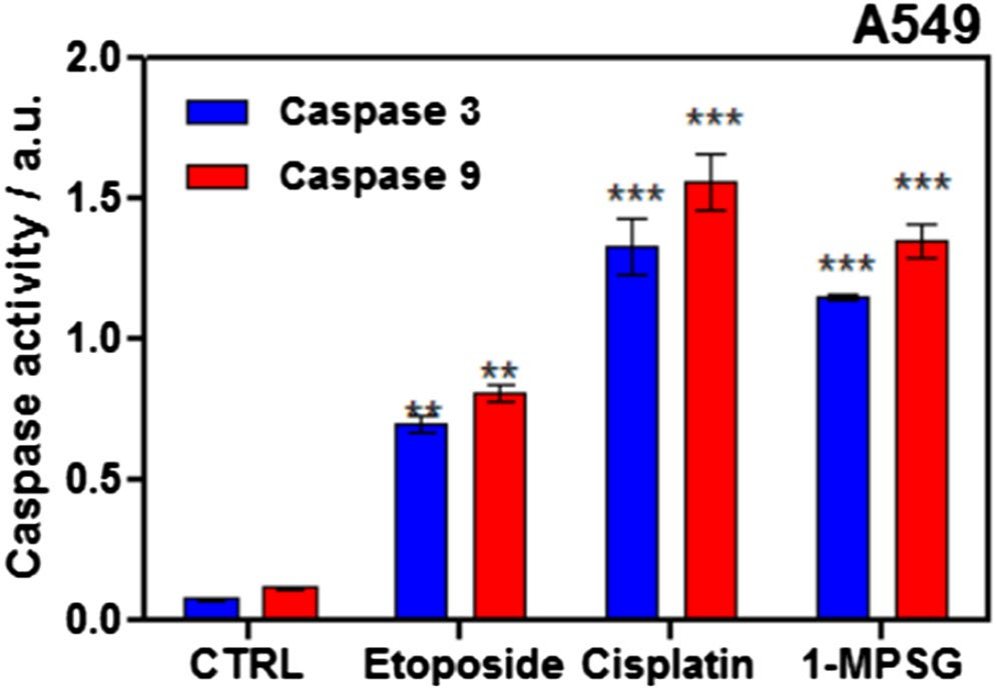
Activation of caspase 3 and caspase 9 in A549 cell line by **1-MPSG**. Etoposide (50 μM) and cisplatin (1 μM) were used as positive controls, while untreated cells constituted control (CTRL). Data are expressed as mean ± SEM, *P < 0.5, **P < 0.1, ***P < 0.01 compared to control. (Two-way ANOVA followed by Bonferroni multiple comparisons test was performed using GraphPad Prism version 5.0.0 for Windows, GraphPad Software, San Diego, California USA, www.graphpad.com.).

It was clearly shown that **1-MPSG** activated both caspase 3 and caspase 9 (the executioner caspase and the initiator caspase, respectively). The measured level of both the caspases after the treatment of A549 cells with **1-MPSG** was much higher in comparison with the control samples (untreated cells without the compound and cells treated with etoposide), as shown in Fig. 5.

**Figure 6:**
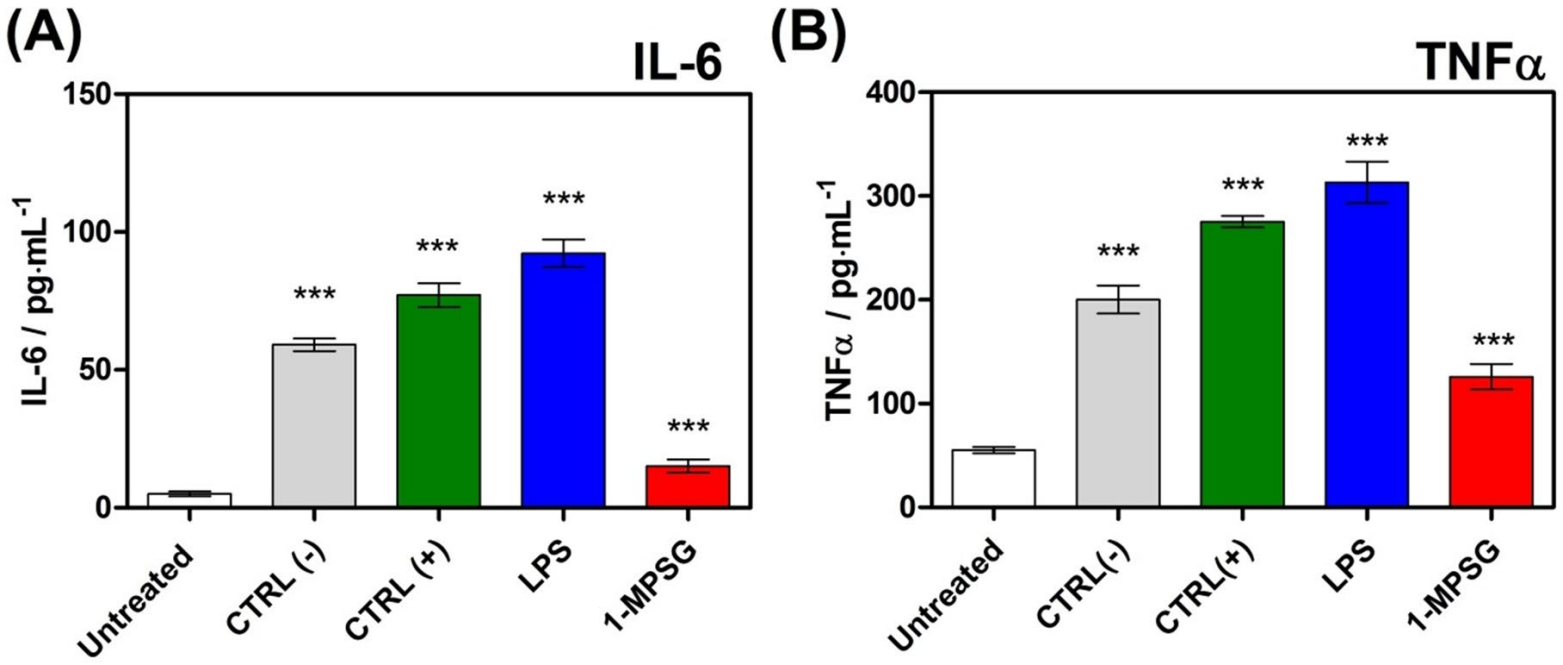
(**A**) IL-6, (**B**) TNF-α production induced by **1-MPSG**, ctrl (-) negative “low” control, ctrl (+): positive “high” control and 0.1 μg/ml LPS. Data are expressed as mean ± SEM, *P < 0.05, **P < 0.01 compared to control CTRL(-). (Two-way ANOVA followed by Bonferroni multiple comparisons test was performed using GraphPad Prism version 5.0.0 for Windows, GraphPad Software, San Diego, California USA, www.graphpad.com.).

The pro-inflammatory effect of MOMP was firstly observed under lack of caspase 9^58,59^. Mitochondrial apoptotic cell death is non-inflammatory in most cases even when MOMP can initiate a plenty of inflammatory signaling pathways. Most probably main reason is that MOMP activates apoptotic caspases to quench inflammation. In order to completely exclude the possibility of pro-inflammatory cytokines activation by the investigated compound, we decided to study the activity of cytokine interleukin-6 (IL-6) and the tumor necrosis factor-alpha (TNF-α). Both mentioned cytokines are extremely important in the pathogenesis of inflammatory disorders^60,61^. The activity of interleukin-6 and TNF-α in the cellular medium after the incubation of A549 cancer cells with/without **1-MPSG** and in the untreated control cells was determined according to the manufacturer’s protocol (Fig. 6).

The incubation of A549 cells with **1-MPSG** resulted in a reduction in IL-6 from *c.a.* 60 pg/ml to *c.a.* 15 pg/ml, while the TNF-α concentration was reduced to 90 pg/ml (the initial concentration: 200 pg/ml). Thus, the studied copper(I) complex inhibited the activity of both interleukins IL − 6 and TNF − α. We did not observe the increase in activation of inflammatory cytokines IL-6 and TNFa what may be explained that cytokine activation only happens when caspases are blocked^62^. Therefore, it can be supposed that the phosphine-peptide conjugate after coordination with the Cu^+^ ion and the complex formation did not cause a possible inflammation, but rather anti-inflammatory properties can be envisaged.

Taking into account the obtained results, it can be postulated that the studied copper(I) complex **1-MPSG** can induce a controlled “cell suicide” (apoptotic death) of A549 cancer cells. This means that **1-MPSG** initiates cell death which is natural and harmless to the body and above all, unlike necrosis, it does not cause any inflammation^41,42^. At the current stage of this research, it can be supposed that the induction of apoptosis in the examined cells probably occurs via the caspase-dependent mitochondrial pathway.

### Generation of reactive oxygen species

**Figure 7:**
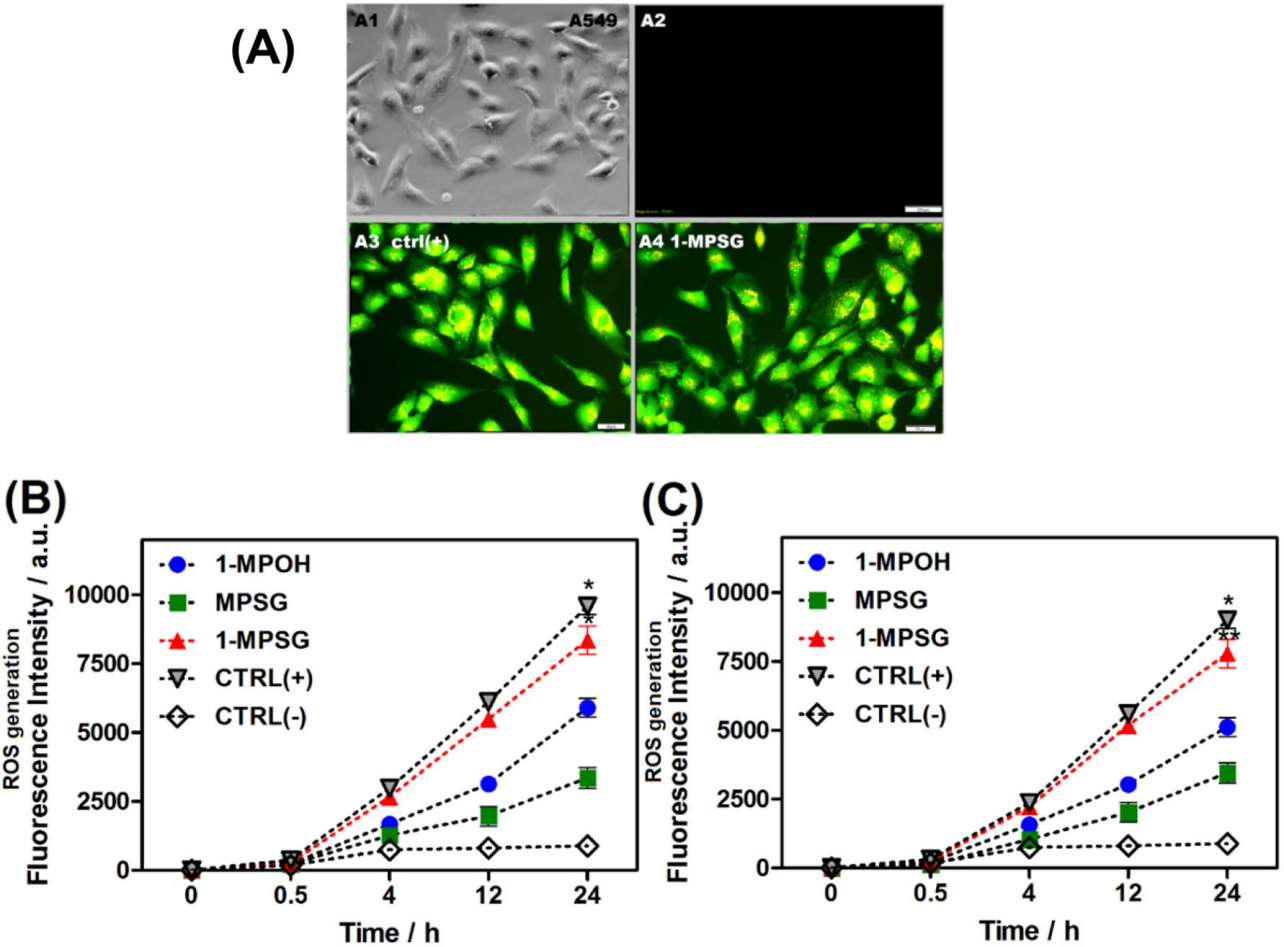
(**A**) Photos of A549 cells after 24 h treatment without and with **1-MPSG** or pyo (pyocyanin) for cyto-ID hypoxia/oxidative stress detection (λex = 505 nm, λem = 524 nm). (**B**) The ROS production in A549 cells after 30 min., 4, 12 and 24 h using H_2_DCF-DA for: **1-MPOH**, **MPSG**, **1-MPSG**, CTRL(+): H_2_O_2_ as positive control and CTRL(-): negative control, cells without compound; (**C**) Oxidative stress induced by the total ROS production in A549 cells after 30 min., 4, 12 and 24 h detected by CYTO-ID Hypoxia/Oxidative Stress Test for: **1-MPOH**, **MPSG**, **1-MPSG**, CTRL(+): pyocyanin as positive control and CTRL(-): negative control. Data are expressed as mean ± SEM, *P < 0.05, **P < 0.01 compared to control CTRL(-). (Two-way ANOVA followed by Bonferroni multiple comparisons test was performed using GraphPad Prism version 5.0.0 for Windows, GraphPad Software, San Diego, California USA, www.graphpad.com.).

Several studies have demonstrated that mitochondrial damage leads to the increased production of reactive oxygen species (ROS)^63^. An elevated ROS level, in turn, may cause a cell cycle arrest or lead to apoptosis^41,42^. Therefore, it was of interest to investigate whether the cellular damage induced by the copper complex would lead to increased ROS generation. Cellular ROS production in A549 cells upon 30 min, 4, 12 and 24 h treatment with **1-MPOH**, **MPSG** and **1-MPSG** (IC_50_) was monitored by a fluorescent H_2_DCF-DA ROS probe (λ_ex_ = 495 nm, λ_em_ = 530 nm). In addition, the level of oxidative stress induced by the total ROS production was determined using a cyto-ID hypoxia/oxidative stress detection kit (λ_ex_ = 505 nm, λ_em_ = 524 nm). H_2_O_2_ and pyocyanin were used as positive controls in the first and second case, respectively. The fluorescence intensity-ROS dependence and oxidative stress concentration over time (four different incubation times: 30 min, 4, 12, and 24 h) are presented in Fig. 7.

It was proved that the investigated complex **1-MPSG** (Fig. 7) exhibited the ability to induce ROS production inside the treated A549 cells. Both the tests showed that the studied complex was able to induce ROS generation at a significantly higher level than pyocyanin (pyocyanin toxicity results from its ability to undergo reduction by NAD(P)H and the subsequent generation of superoxide and H_2_O_2_)^64^ and than **MPSG** and **1-MPOH** (the copper complex without the peptide motif). Moreover, it was observed that **1-MPSG**-mediated cytotoxicity; copper uptake and the ROS level inside A549 cancer cells increase with treatment time (vide supra, Fig. 1 and 7). This suggests that ROS are probably primarily involved in the observed cytotoxicity and in the mechanism of cell death. More precise experiments will help to explain their remarkable contribution to the observed cytotoxicity (vide infra).

In order to determine the effect of different concentrations of **1-MPSG** on the production of intracellular ROS we used an H_2_DCFH-DA probe and flow cytometry analysis (Figs. 11, S14 and S15, Supporting Information). A549 cells were exposed to **1-MPSG** at various concentrations for 24 h and the overall level (intracellular and mitochondrial) of ROS was measured throughout the exposure time.

**Figure 8:**
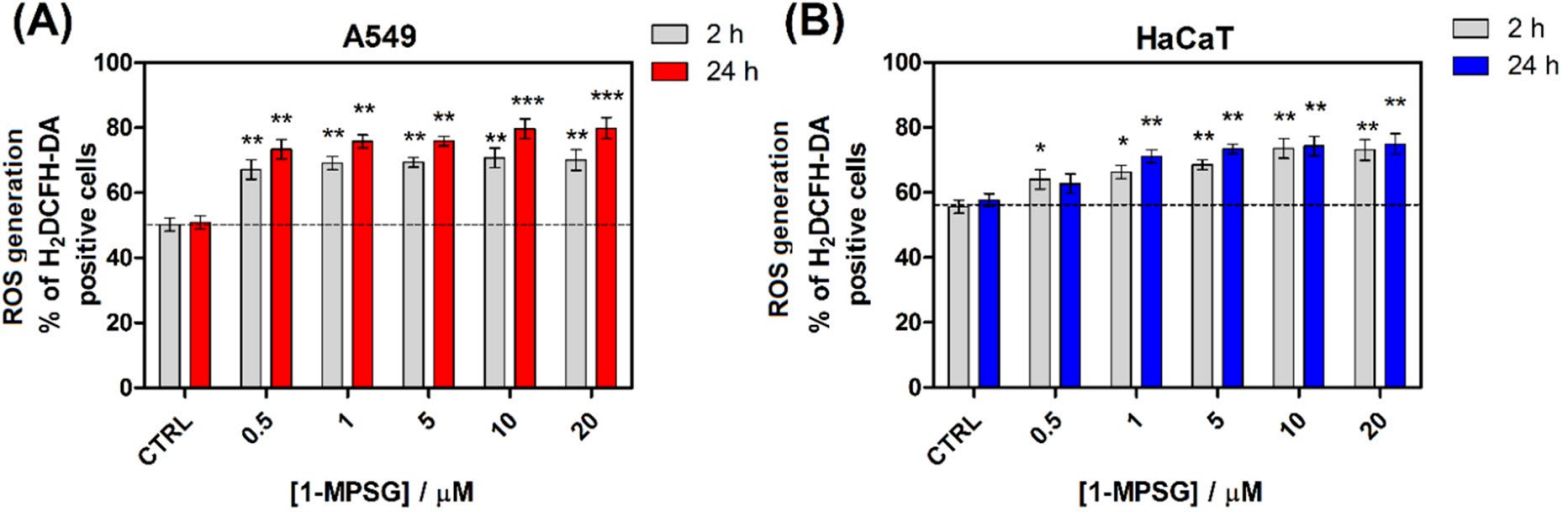
Effect of **1-MPSG** on the intracellular ROS generation of (**A**) A549 and (**B**) cells. A549 cells were incubated with various concentrations of **1-MPSG** for 24 h. ROS generation was determined using an H_2_DCF-DA probe. Data are presenting as analysis of flow cytometry histograms with the average H_2_DCF-DA fluorescence intensity. Data are expressed as mean ± SEM, *P < 0.05, **P < 0.01, ***P < 0.001 compared to control. (Two-way ANOVA followed by Bonferroni multiple comparisons test was performed using GraphPad Prism version 5.0.0 for Windows, GraphPad Software, San Diego, California USA, www.graphpad.com.).

It was demonstrated that ROS generation in the A549 cells treated with **1-MPSG** significantly increases over time in a concentration-dependent manner. Using the H_2_DCFDA probe reacting with multiple ROS species in the cells, a substantial increase in intracellular ROS was observed after exposure to the **1-MPSG** concentration of 0.5 μM, continuously increased up to 20 μM. In comparison with the untreated control cells, a concentration-dependent increase in H_2_DCF-DA positive cells after the treatment with **1-MPSG** was observed. As shown in Fig. 8, the level of intracellular ROS in A549 cells treated in the concentration range of 0–20 μM **1-MPSG** was augmented from 50 to 70% after 2 h, and to 80% after 24 h of incubation, respectively. These values are significantly higher than for the control group, indicating cell death dependence on the increasing level of oxidative stress. However, in the case of HaCaT cells (Fig. 8B), the less significant increase in ROS generation, as well as less time-dependency of their production, may be observed. In both tested time points, ROS generation increases reaching about 20% compared to untreated control.

**Figure 9:**
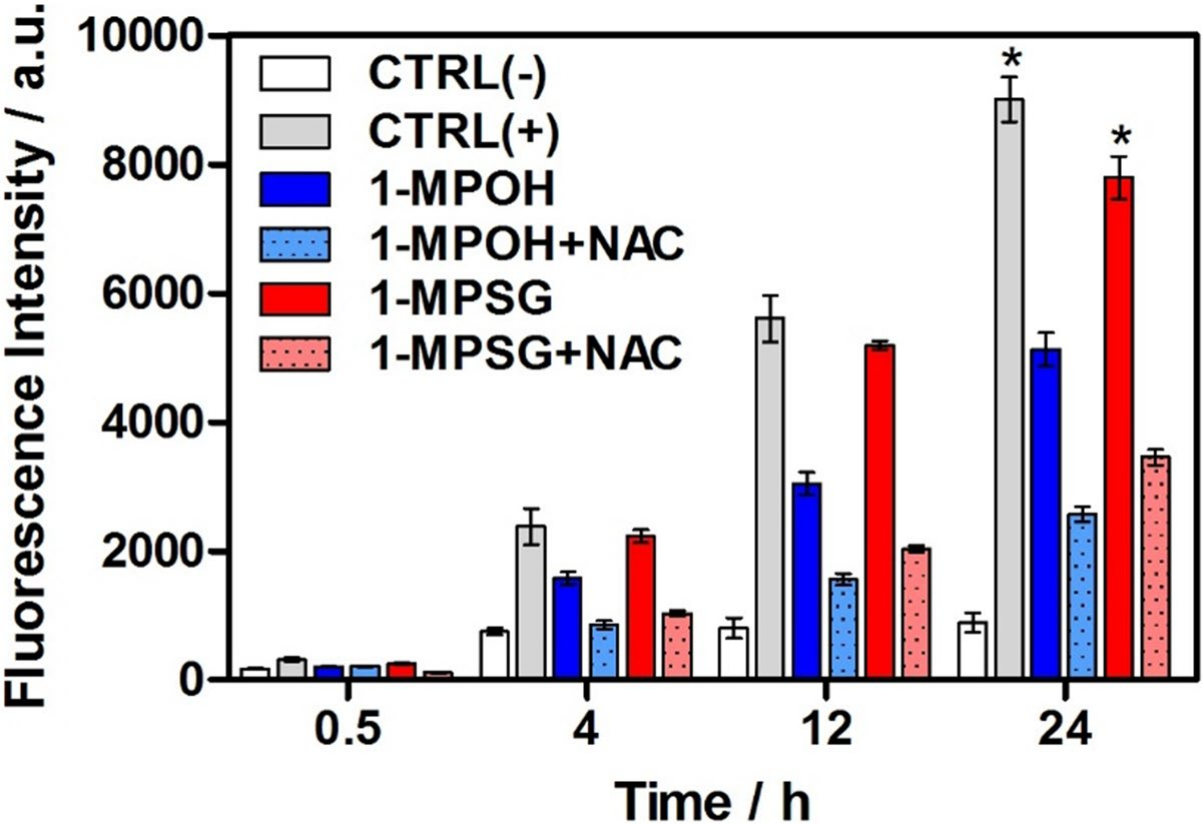
Changes in fluorescence intensity for A549 cells incubated with **1-MPOH, 1-MPSG** at c = 1 µM in presence and absence of NAC (5 mM). The results were obtained after 30 min, 4, 12, and 24 h of incubation using H_2_DCF-DA probe. CTRL(-): as a negative control, cells without copper compounds; CTRL(+): H_2_O_2_. *P < 0.05, compared to control (CTRL(-)). (Two-way ANOVA followed by Bonferroni multiple comparisons test was performed using GraphPad Prism version 5.0.0 for Windows, GraphPad Software, San Diego, California USA, www.graphpad.com.).

It is noteworthy that a decrease in A549 cell viability was observed and this change correlated with a decrease in MMP. The above results support the hypothesis that **1-MPSG** is able to noticeably depolarize mitochondrial potential in A549 cells, involving the time-dependent ROS generation process. In order to confirm ROS generation, the N-acetyl derivative of the amino acid L-cysteine (a NAC and ROS scavenger) was used as a direct exogenous antioxidant (Fig. 9). ROS scavengers and especially NAC are commonly used to confirm the involvement of ROS in drug-induced apoptosis. It was reported several times that NAC addition fully abolished ROS-dependent cell death induced by *e.g.* H_2_O_2_^65–68^.

NAC has a free thiol group aiding in free radical scavenging and can also act as a precursor of reduced glutathione (GSH)—a well-known antioxidant^64^. It is noteworthy that significant inhibition in ROS generation was observed in cells pre-treated with the N-acetyl L-cysteine thiol antioxidant (5 mM) and then treated with **1-MPOH** and **1-MPSG**. This confirms not only ROS generation inside the studied cells, but also the hypothesis about the significant role of ROS in the induction of caspase-dependent mitochondrial apoptotic pathway caused by the Cu(I) complex, bearing in mind the effective accumulation in mitochondria.

### 1-MPSG arrests cell cycle in A549 cells

**Figure 10:**
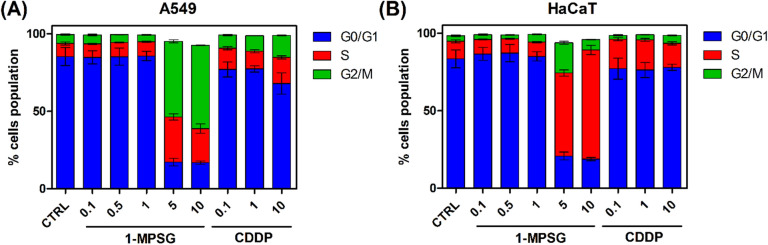
Flow cytometry was used to determine the cell cycle distribution of (**A**) A549 and (**B**) HaCaT cells treated with **1-MPSG** and cisplatin (CDDP). Data are presented as an analysis of the cell cycle distribution of cells after 1-MPSG and CDDP treatment. Data are expressed as mean ± SEM. (The figure was plotted using GraphPad Prism version 5.0.0 for Windows, GraphPad Software, San Diego, California USA, www.graphpad.com.).

In order to determine whether **1-MPSG** affects the A549 and HaCaT cells cycle, its distribution was assessed by flow cytometry and compared to cisplatin (CDDP, a well-known reference chemotherapeutic drug). **1-MPSG** caused a cell-cycle arrest in a dose-dependent manner in the A549 and HaCaT cells (Figs. 10, S16 and S17, Supporting Information).

Our data showed that 5 and 10 μM **1-MPSG** could suppress cell growth and resulted in a remarkable accumulation of cells in G2/M accompanied by a decrease in G0/G1 DNA content. After the exposure of A549 cells to 10 μM **1-MPSG** for 24 h, the percentage of cells in the G2/M phase increased to 53.7% (Fig. 10). It is suggested that when cell cycle arrest occurs in cell division, the DNA damage and error are difficult to repair^69^. Cell cycle arrest at the G2/M phase indicates that the damage of intracellular DNA is difficult to repair. The G2/M phase arrest and subsequent apoptosis induction in vitro were also reported for #2714—a compound with high potential anti-lung cancer efficacy^70^. Treatment of the non-cancerous cells with 5 and 10 μM **1-MPSG** resulted in a significant increase in the percentage of cells in the S phase (70.4%) than untreated cells (11.3%). In both cell lines, cisplatin resulted in a slight increase in the S phase population. However, in HaCaT, this effect was observed at lower concentrations (from 0.1 μM) than in A549 cells (10 μM). Moreover, in the case of A549, cisplatin leads also to an increase in cell population in the G2/M phase (8.5%, 10%, and 14.1% for 0.1, 1, and 10 μM cisplatin, respectively). Representative flow cytometric graphs of cells treated with different concentrations of **1-MPSG** and cisplatin for 24 h are shown in Figs. S16 and S17 (Supporting Information).

### 3D A549 tumor spheroid model

**Figure 11:**
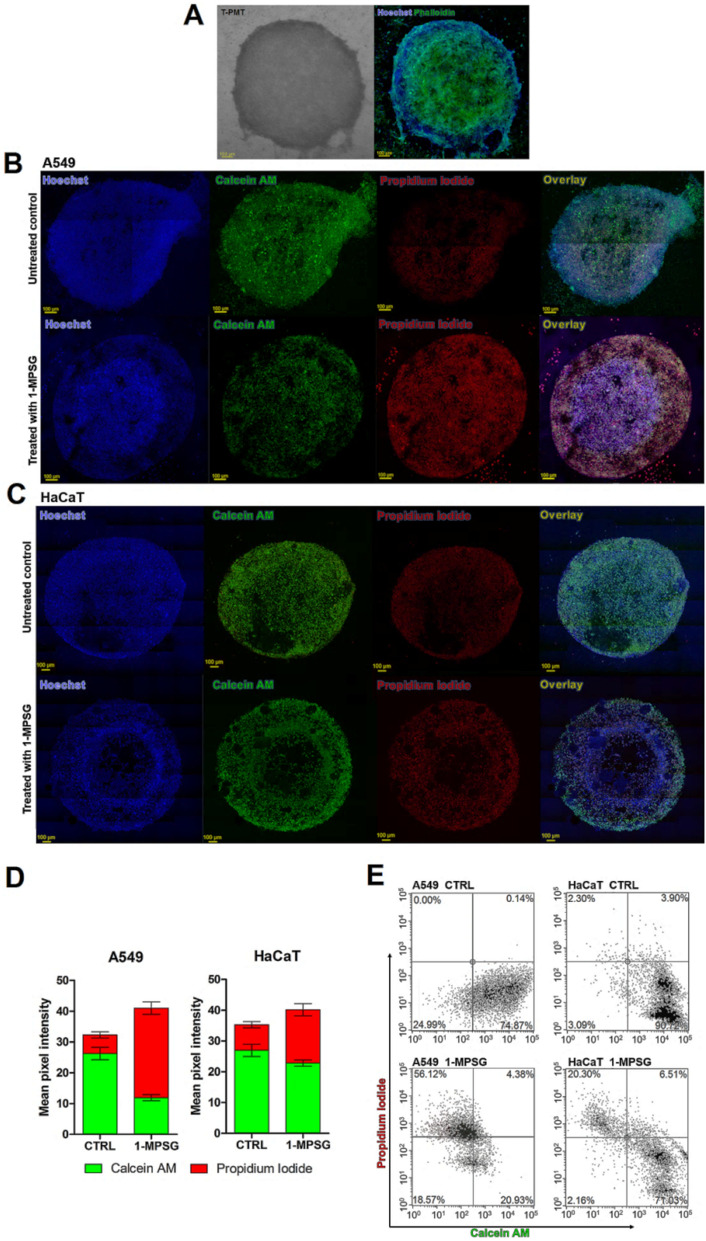
(**A**) 3D A549 spheroid model—bright-field confocal image of representative spheroid (left) and the same spheroid stained with Hoechst33342 (blue) and phalloidin-FITC (green) (right); (**B**) **1-MPSG** cytotoxicity in A549 and (**C**) HaCaT spheroids—representative live/death fluorescence images of spheroids subjected to 10 μM concentration of **1-MPSG** and corresponding control untreated spheroids grown in the same culturing conditions; (The figure was plotted using GraphPad Prism version 5.0.0 for Windows, GraphPad Software, San Diego, California USA, www.graphpad.com.) (**D**) Live/dead spheroids staining—quantification of pixel intensity from confocal images. Data expressed as mean ± SEM); (**E**) Live/dead analysis carried out on spheroids using flow cytometry (spheroids were stained with Propidium iodide and Calcein AM).

Encouraged by the preliminary results in the 2D cell culture model, we applied herein the 3D technology. The latter has received much attention mainly owing to the fact that it provides a more accurate model of the in vivo conditions mimicking the solid tumor growth environment. Therefore, the 3D model of human lung adenocarcinoma (A549) spheroids was chosen for further study. At the same time the research was continued on the healthy cell line (HaCaT) as a reference line (Figs. 11, S18 and S19, Supplementary Material). The therapeutic potential of the synthesized **1-MPSG** compound towards 3D spheroids was demonstrated by simultaneous in situ Live/Dead fluorescence staining, providing the spatial distribution of dead cells and cytotoxicity information (Fig. 11). **1-MPSG** significantly reduced the viability of 3D A549 spheroids, with dead cells distributed across the inner core of the spheroids. Indeed, when exposed to the drug (10 μM), the structural spheroidal integrity was destroyed. In the case of the untreated 3D spheres, they retained their normal morphology and structure and dead cells were observed, as expected, mainly in the superficial regions of the spheroids.

The relative level of Calcein AM and PI-positive cells was assessed by image analysis and expressed as green/red mean pixel intensities (Fig. 11D). While in the case of non-cancerous HaCaT cells, a much less prominent effect on the necrotic destruction of the sphere was observed (Fig. 11B–D). These significant differences in the observed selective activity of **1-MPSG** regarding cancer A549 cells while the limited effect toward normal HaCaT cells have also been demonstrated using flow cytometry. Figure 11E shows the flow cytometry analysis results on the cells from 3D spheroid culture under **1-MPSG** treatment. The dot plots are drawn with green fluorescence intensity (Calcein AM) as the x-axis and red fluorescence intensity (PI) as the y-axis. Therefore, the live cells appear at the lower right corner in the plots, and the dead cells appear at the upper left corner. All the control experiments showed more than 80% of the cells are alive. In the **1-MPSG** treated spheroids, reasonable cytotoxicity can be observed, leading to 51% of dead cells in A549 spheroids and 20% in HaCaT spheroids, respectively. It clearly shows the selective cytotoxic potential of the copper(I) complex with peptide-phosphino conjugate.

## Conclusions

Copper-based compounds are promising entities for target-specific next-generation anticancer drugs. Some of them were reported as promising anticancer agents capable of induction of apoptosis with the mitochondrial-controlled pathway. Recently, we described the synthesis, comprehensive characterization and preliminary biological activity of the series of copper complexes against various cancer cell lines with respect to commercially used metal-based anticancer drug cisplatin. We indicated that the introduction of methoxy group onto the phenyl rings of the phosphine ligand coordinated to the copper(I) ion resulted in a relevant increase of cytotoxicity in selected cancer cell lines. Moreover, we have highlighted that attachment of a peptide carrier significantly increased the selectivity towards cancer cells. In agreement with our previous in vitro data, herein, we confirmed in the 3D-culture model that [CuI(2,9-dimethyl-1,10-phenanthroline)P(*p*-OCH_3_-Ph)_2_CH_2_SarcosineGlycine] (**1-MPSG**) selectively inhibits cell growth of human lung cancer cells, when compared with normal somatic cell line (MRC5, HEK293T, and in particular HaCaT). Our investigation on elucidation of action of the studied herein copper(I) complex with phosphine-peptide conjugate (**1-MPSG**) allowed to formulate the following general conclusions, also adequate for other studied by us Cu(I) compounds. Low-toxic, synthesized Cu(I) compound is effectively taken up by A549 cells and exhibits obvious organelle accumulation. The redox-active **1-MPSG** mediates intracellular ROS alterations in A549 and HaCaT cells. A considerable upsurge in intracellular ROS and a significant reduction of MMP level were determined in the **1-MPSG**-treated A549 cells. It was also observed **that 1-MPSG** considerably augmented the ROS levels compared to the control and the increase was significantly higher than for non-cancerous cells. The results suggest that investigated Cu(I) compound may trigger apoptosis also through ROS generation. Another reason for apoptosis might be the observed capacity of **1-MPSG** to cause cell G2/M cycle arrest of A549 cells in a dose-dependent manner. Both the activation of caspases-3/9 and the decrease of mitochondrial membrane potential confirm the apoptotic pathway of cell death. The activity of caspases, and in consequence, triggered apoptosis in **1-MPSG**-mediated cell death was also confirmed using an assay with Z-VAD-FMK caspases inhibitor. Furthermore, we did not observe the increase in activation of inflammatory cytokines IL-6 and TNFa what may be related to the fact that these cytokines activation only happens when caspases are blocked (via NFkB or cGAS/STING). Thus, it can be supposed that phosphine-peptide conjugate after coordination with Cu^+^ ion and the complex formation did not cause possible inflammation, but rather anti-inflammatory properties can be envisaged. In final, the cytotoxicity of **1-MPSG** was also confirmed in A549 3D tumor spheroids model, which may be a more predictive and accurate preclinical model than 2D cultures. These data help us to develop further and understand the efficacy of synthesized anticancer metallo-complexes in more physiologically relevant models.

To sum up, our findings reveal that the caspase-dependent mitochondrial pathway is, at least partially, involved in the mode of **1-MPSG** copper(I) complex cytotoxic action. All demonstrated experimental evidences, in particular examined 3D spheroidal model, indicate a presumable molecular mechanism different from DNA targeting dominating in Pt(II) drugs, as in the case of well-known cisplatin. This proves that **1-MPSG** can be considered as a potential selective anticancer drug of the new generation.

## Experimental section

### Materials

An atmosphere of dry oxygen-free dinitrogen was applied during all syntheses and operations due to application of standard Schlenk techniques or a glove box^36^. SarGly peptide was purchased from Bachem (Switzerland), while HP(*p-*OCH_3_*-*Ph)_2_, dmp, CuI, other chemicals and solvents from Sigma-Aldrich (Germany). The latter reagents were used without further purification, while solvents were deaerated before use^36^.

### Synthesis

The compounds **MPOH**, **MPSG**, **MPSG**, **1-MPSG** were prepared and characterized according to methods described by us previously^36^.

### Cell cultures

Cell lines: MCF7 (human breast adenocarcinoma, morphology: epithelial-like, ATCC: HTB-22), A549 (human lung adenocarcinoma, morphology: epithelial, ATCC: CCL-185), PANC-1 (human pancreatic/duct carcinoma, morphology: epithelial, ATCC: CRL-1469), and HaCaT (human keratinocyte, ATCC) were cultured in Dulbecco's Modified Eagle's Medium (DMEM, Corning) with phenol red, supplemented with 10% fetal bovine serum (FBS) and with 1% streptomycin/penicillin. While, cell lines: DU-145 (human prostate carcinoma, derived from metastatic site: brain, ATCC: HTB-81), MCR-5 (primary line of human pulmonary fibroblasts, ATCC: CCL-171), and HEK293T (human embryonic kidney, ATCC: CRL-3216) were cultured in minimum essential medium (MEM, Corning) with only 10% fetal bovine serum (FBS)^41,42,71^. All media and other ingredients were purchased from ALAB, Poland. All cultures were incubated under a humidified atmosphere with 5% CO_2_ at 37 °C.

### Copper uptake

Cells A549, MCF7, PANC-1, MRC5, HEK293T and HaCat at density of 2 × 10^6^ cells/2 mL were seeded on 6-well plates and incubated with complex **1-MPOH, 1-MPSG** (c = 1 μM for 24 h) at standard conditions (37 °C, 5% CO_2_). Solution of the studied compound was removed; the cells were washed twice with PBS buffer and trypsinized. Cells, for ICP-MS analysis, were mineralized in 1 mL of 65% HNO_3_. Measurement of the concentration of silver ions was determined by a mass spectrometer (ELAN 6100 Perkin Elmer) with an inductively coupled plasma (ICP-MS). Protein content was assessed with Bradford Protein Assay (Thermo Fisher Scientific, Waltham, Massachusetts, USA)^41,42^. The copper content under each condition is expressed as ng Cu/mg protein. The experiment was repeated at least 3 times and results are presented as mean value + S.D.

### Confocal laser scanning microscopy

The intracellular uptake of **1-MPSG** was studied in the A549 cancer cells according to previously applied protocol^72^. In brief, before imaging, A549 cells were seeded on microscopic slides at a density of 1 × 10^5^ cells. Cells were kept for 24 h at 37 °C in a 95% atmospheric air and 5% CO_2_ humidified atmosphere. After being washed with fresh medium, the cells were incubated in the dark with 1 μM solution of **1-MPSG** prepared growth medium for 2 h. Next, after being washed with HBSS, the cells were incubated with specific intracellular organelle probes: 100 nM Mito-Tracker green, 1 µM ERTracker green, 1 μM LysoTracker green (Molecular Probes, Invitrogen Life Technologies; Thermo Fisher Scientific, Waltham, Massachusetts, USA), diluted in HBSS buffer. In addition, cells were incubated for 10 min with Hoechst 33,342. After 30 min incubation, at 37 °C, in the dark, the cells were washed with HBSS two times, and the slide was transferred to the microscope stage and cells were visualized under a confocal microscope Zeiss LSM 880 (Carl Zeiss, Jena, Germany) with a 63 × oil immersion objective. Images were analyzed by Zeiss ZEN Software. The Pearson colocalization coefficient was calculated using Fiji Image J software.

### Cell death analysis by flow cytometry

Annexin V Apoptosis Detection Kit FITC (Invitrogen) and Propidium Iodide (Thermo Fischer Scientific, Waltham, Massachusetts, USA) were used to distinguish cell death (apoptotic and necrotic cells) induced by studied compounds quantitatively due to previously described protocol^41,42^. In brief, the studied compounds **1-MPSG** (in a broad range of concertation ranging between 100 and 0.01 µM) were incubated for 24 h with A549 and HaCaT cells (seeded at density 5 × 10^5^ cells/mL) in 12-well plates. After this time, the compound solutions were removed, and the cells were washed twice with PBS buffer (phosphate-buffered saline, pH = 7.4). Trypsin was added to the cells and then they were left for 10 min at 37 °C in a humidified atmosphere containing 5% CO_2_. The cells were collected, centrifuged, and separated from the supernatant, then washed twice with 0.5 ml PBS buffer (buffer phosphate saline NaCl, KCl, Na_2_HPO_4_, KH_2_PO_4_) and suspended in Binding Buffer. Fifteen minutes before measuring, cells were stained with Annexin V-FITC and PI and incubated in the dark. Viable and dead (early apoptotic, late apoptotic, and necrotic) cells were detected using the BDAccuri flow cytometer (BD Biosciences). The experiment was repeated at least 3 times.

### Effect of caspase inhibitors on 1-MPSG induced apoptosis in A549 and HaCaT cells

Cells were preincubated for 2 h with the 20 μM Z-VAD-FMK (Calbiochem, Merck) and treated with 1 μM **1-MPSG** for 24 h. Then cells were harvested and analyzed by flow cytometry using AnnexinV-FITC and propidium iodide (Invitrogen) accordingly to the procedure described above. The experiment was repeated at least 3 times.

### Detection of mitochondrial membrane potential (ψ)

Mitochondrial membrane potential (MMP) depletion was determined by JC-10 Assay (Life Technologies, USA). A549 and HaCaT cells were seeded on 96-well plates at 1 × 10^4^ cells/0.2 mL. After 24 h, the medium was replaced with solutions of **MPSG**, **1-MPOH**, **1-MPSG** at IC_50_ concentration, gentamicin (0.5 mg/mL), and ciprofloxacin (10 µg/mL) as the positive and negative control, respectively. After that, cells were incubated for 24 h at standard condition (37 °C, 5% CO_2_). Then, they were washed twice with PBS buffer and incubated with JC-10 for 1 h. Afterwards, emission was measured at two different excitation wavelengths (λ_exc_ = 540 nm, λ_em_ = 570 nm) and (λ_exc_ = 485 nm, λ_em_ = 530 nm). Results are presented as the intensity ratio of red to green emission (mean + S.D.)^41,42^.

### Caspase activity assays

Colorimetric Protease Caspase-3/CPP32 and Caspase-9/Mch6/Apaf-3 Colorimetric Protease kits (Life Technologies, USA) were used to detect activated caspases 3 and 9, respectively. The effect of compounds on the activation of caspase 3 and 9 in A549 cells was monitored spectrophotometrically using the substrates DEVD-pNA and LEHD-pNA, respectively, according to the supplier's instructions. In brief, cells at 5 × 10^5^ cells/2 mL were seeded on 6-well plates and incubated for 24 h. Then, after medium aspiration cells were treated at 1 μM for 24 h with copper(I) complex (**1-MPSG**), 1 μM CDDP and etoposide (positive control, 50 μM) at standard condition (37 °C, 5% CO_2_). Afterward, cells were trypsinized, centrifuged, and suspended in 50 μL of the cooled cell lysis buffer (Cell Lysis Buffer) for 10 min. The cell degradation products were centrifuged (1 min, 10 000 × g) and the supernatant was transferred to fresh Eppendorf tubes and left on ice. The concentration of isolated proteins was determined in all lysates using Bradford Protein Assay (Thermo Fisher Scientific; Waltham, Massachusetts, USA)^36^. 50 μL of 10 mM dithiothreitol (DTT) solution in reaction buffer and 5 μL of 4 mM DEVD-pNA (in the case of caspase 3) or LEHD-pNA (in the case of caspase 9) were added to 1 mg/mL of collected proteins and incubated for 2 h in the dark at 37 °C. Absorbance in 96-well plates at 400 nm was measured using a plate reader (200 M PRO NanoQuant; Tecan, Switzerland) (free p-NA). Samples were analyzed in triplicate, and standard deviations were calculated.

### Inflammatory activity

IL6 and TNF alpha were quantified in the conditioned cell culture medium using the Human IL6 ELISA Kit and Human TNF alpha ELISA, respectively, according to the manufacturer's protocol (Immuniq, Poland) using Microplate Readers (Tecan). Negative “low” control ctrl (-), positive “high” control ctrl (+) and 0.1 μg/ml LPS were provided and described in manufacturer protocol provided by producers. Experiments were performed at least three times.

### Reactive Oxygen Species generation

Production of Reactive Oxygen Species (ROS) in A549 and HaCaT cells induced by **MPSG**, **1-MPOH**, **1-MPSG** photometric tests using: 5-(and-6)-chloromethyl-20,70-dichlorodihydrofluorescein, diacetate acetyl ester (H_2_DCF-DA), and a Cyto-ID Hypoxia/Oxidative Stress Detection Kit according to the procedure described elsewhere^36^. Pyocyanin or H_2_O_2_ and untreated cells were used as positive and negative controls, respectively. The influence of N-acetylcysteine antioxidant (NAC) was checked. All experiments were carried out following the procedures described in our previous papers^73^.

### Fluorescence microscopy

Oxidative stress was detected by staining with Cyto-ID Hypoxia/Oxidative Stress Detection Kit for 10 min and examined using a fluorescence inverted microscope (Olympus IX51, Japan) with an excitation filter 470/20 nm. Photographs of cells after treatment with the tested compounds were taken under magnification 20 × .

### *ROS detection *in vitro

Intracellular ROS levels were measured in a 12-well plate format using the H_2_DCFDA probe. A549 and HaCaT cells (30,000/well) were seeded in the 12-well black-wall plate overnight before the experiments. The following day, cells were washed with HBSS and treated with **1-MPSG** at various concentrations (0.5–20 μM) for 24 h. After this incubation, cells were detached with accutase and then, the staining solution (10 μM of H_2_DCFDA in HBSS) was added to each sample, and cells were incubated for 40 min at 37 °C. The cells were then washed twice with HBSS, and HBSS was added to each sample. Then, all samples were analyzed by flow cytometry using BD Accuri Flow Cytometer.

### Cell cycle analysis

The A549 and HaCaT cells (3 × 10^5^/well) were seeded in 12-well plates and treated with various concentrations of **1-MPSG** and cisplatin (CDDP) for 24 h. Synchronization of A549 and HaCaT cell cultures was performed by serum starvation. Serum starvation is widely used for synchronizing donor cells by arresting them in the G0/G1 phase of the cell cycle^74,75^. Cell cultures were seeded and incubated in a growth medium with 20% FBS overnight to synchronize the cell cultures. Then the cultures were rinsed by PBS and changed to serum-free medium. After serum starvation for 18 h, the cells were passaged and released into the cell cycle by the addition of serum. Then cells were treated with **1-MPSG** and CDDP for 24 h. For FACS analysis, cell samples were harvested with trypsinization and stained with propidium iodide (20 ug/mL). Cell cycle phase distributions were analyzed by flow cytometry (BD Bioscience). Experiments were reaped at least three times.

### Three-dimensional culturing in vitro

A549 or HaCaT cells were grown in DMEM (high glucose) supplemented with 10% heat-inactivated fetal bovine serum and 1% antibiotics. For 3D cell culture, the GelTrex matrix was used as a basement membrane, which has an appropriate gel structure and established biological activity to promote the growth and differentiation of various cells. To prepare 3D cultures by the hanging-drop method, 50 μL volumes of cells in the single-cell suspension of 50,000 cells/mL were used. When the cells formed spheroids, they were plated on the GelTrex matrix and were allowed to adhere before adding a complete growth medium with 2% GelTrex. All cultures were maintained in an incubator at 37 °C in an atmosphere of 5% CO_2_^76^.

### Assessment of cytotoxic effect in the 3D culture model

To characterize the morphology, spheroids were fixed in 3.7% formaldehyde in PBS at room temperature for 20 min. Then, spheroids were washed two times with PBS and incubated with 0.1% Triton X-100 in PBS i for 3–5 min to increase permeability. Then cells were washed 3 times in PBS and incubated with Phalloidin-FITC (Thermo Fisher Science, Waltham, Massachusetts, USA) at working solution at room temperature for 90 min and stained with 10 µg/mL Hoechst33342 (Invitrogen) in the same time. After incubation, cells were rinsed 3 times, preserved in mounting medium and imaged using confocal microscopy with z-stack mode. To visualize the cytotoxicity of **1-MPSG** against A549 and HaCaT spheroids, the live/dead staining with Hoechst 33342, Calcein AM and propidium iodide was performed. After the treatment with **1-MPSG**, media was removed from culture dishes of 3D spheroids by gentle aspiration followed by a wash with PBS with Ca^2+^ and Mg^2+^ and incubation at 37 °C for 40 min with 2 μM calcein AM, 10 μg/mL Hoechst 33,342 and 2 μM propidium iodide diluted in PBS before imaging. The addition of reagents to each culture dish was staggered such that the incubation period was exactly 40 min for each dish. Then spheroids were washed with PBS and imaged with a confocal microscope Zeiss LSM 880 (Carl Zeiss, Jena, Germany) with a 10 × objective. Images were analyzed by Zeiss ZEN Software.

### Flow Cytometry analysis for spheroids

In order to perform the flow cytometry analysis on spheroids treated with **1-MPSG** (10 uM, 24 h), the spheroids (N = 10) were dissociated into single cells using trypsin–EDTA 0.25% for 5 min with gentle pipetting up and down to minimize aggregated cell population for the following analysis. The single-cell suspension from the dissociated spheroids was then incubated with 5 μM Calcein AM (live stain) for 30 min at room temperature, protected from light, and for 15 min with Propidium iodide (dead stain) in HBSS. The stained cells were analyzed by a flow cytometer (BDAccuri, BD Bioscience) measuring green fluorescence emission for Calcein AM and red fluorescence emission for PI.

### Statistical analysis

Results are present as mean ± standard error of the mean (SEM) from at least three independent experiments. Statistical significance was determined by one-way or two-way ANOVA with Bonferroni post hoc test using GraphPad Prism version 5.0.0 for Windows, GraphPad Software, San Diego, California USA, www.graphpad.com.).

## Supplementary Information

Supplementary Information.
