# Supplementary material for: Evaluation of anticancer activity in vitro of a stable copper(I) complex with phosphine-peptide conjugate
Source: Sci Rep. 2021 Dec 14;11:23943. doi: 10.1038/s41598-021-03352-2 (PMC8671550; doi:10.1038/s41598-021-03352-2)
Supplement: Supplementary file 1 — Supplementary Information. [file 41598_2021_3352_MOESM1_ESM.docx]

Evaluation of anticancer activity *in vitro* of a stable copper(I) complex with phosphine-peptide conjugate.

**Urszula K. Komarnicka,^a*^ Barbara Pucelik,^b*^ Daria Wojtala,^a^ Monika Lesiów,^a^ Grażyna Stochel,^c^ Agnieszka Kyzioł^c*^**

*^a^Faculty of Chemistry, University of Wroclaw, Joliot-Curie 14, 50-383 Wroclaw, Poland.*

*^b^Małopolska Center of Biotechnology, Jagiellonian University, Gronostajowa 7A, 30-387, Kraków, Poland.*

*^c^Faculty of Chemistry, Jagiellonian University, Gronostajowa 2, 30-387, Kraków, Poland.*

**Corresponding authors: urszula.komarnicka@chem.uni.wroc.pl, barbara.pucelik@uj.edu.pl and kyziol@chemia.uj.edu.pl*

**ABSTRACT**

**[CuI(2,9-dimethyl-1,10-phenanthroline)P(p-OCH_3_-Ph)_2_CH_2_SarcosineGlycine] (1-MPSG), highly stable in physiological media phosphino copper(I) complex – is proposed herein as a viable alternative to anticancer platinum-based drugs. It is noteworthy that, 1-MPSG significantly and selectively reduced cell viability in a 3D spheroidal model of human lung adenocarcinoma (A549), in comparison with non-cancerous HaCaT cells. Confocal microscopy and an ICP-MS analysis showed that 1-MPSG effectively accumulates inside A549 cells with colocalization in mitochondria and nuclei. A precise cytometric analysis revealed a predominance of apoptosis over the other types of cell death. In the case of HaCaT cells, the overall cytotoxicity was significantly lower, indicating the selective activity of 1-MPSG towards cancer cells. Apoptosis also manifested itself in a decrease in mitochondrial membrane potential along with the activation of caspases-3/9. Moreover, the caspase inhibitor (Z-VAD-FMK) pretreatment led to decreased level of apoptosis (more pronouncedly in A549 cells than in non-cancerous HaCaT cells) and further validated the caspases dependence in 1-MPSG-induced apoptosis. Furthermore, the 1-MPSG complex presumably induces the changes in the cell cycle leading to G2/M phase arrest in a dose-dependent manner. It was also observed that the 1-MPSG mediated intracellular ROS alterations in A549 and HaCaT cells. These results, proved by fluorescence spectroscopy and flow cytometry, suggest that investigated Cu(I) compound may trigger apoptosis also through ROS generation.**


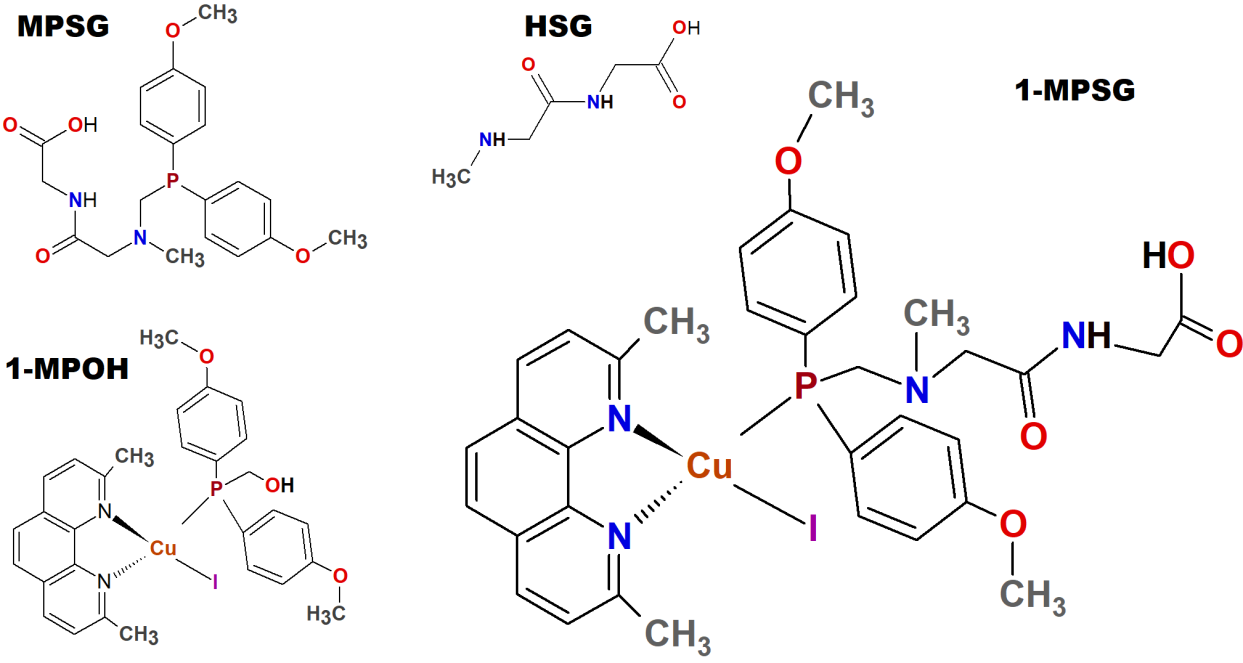


**Figure S1.** Schematic view of the chemical structure of peptide sar-gly (**HSG**), phosphine derived from peptide sar-gly (**MPSG**), copper(I) complexes without peptide motif (**1-MPOH**) and copper(I) complex with phosphino-peptide conjugate (**1-MPSG**).


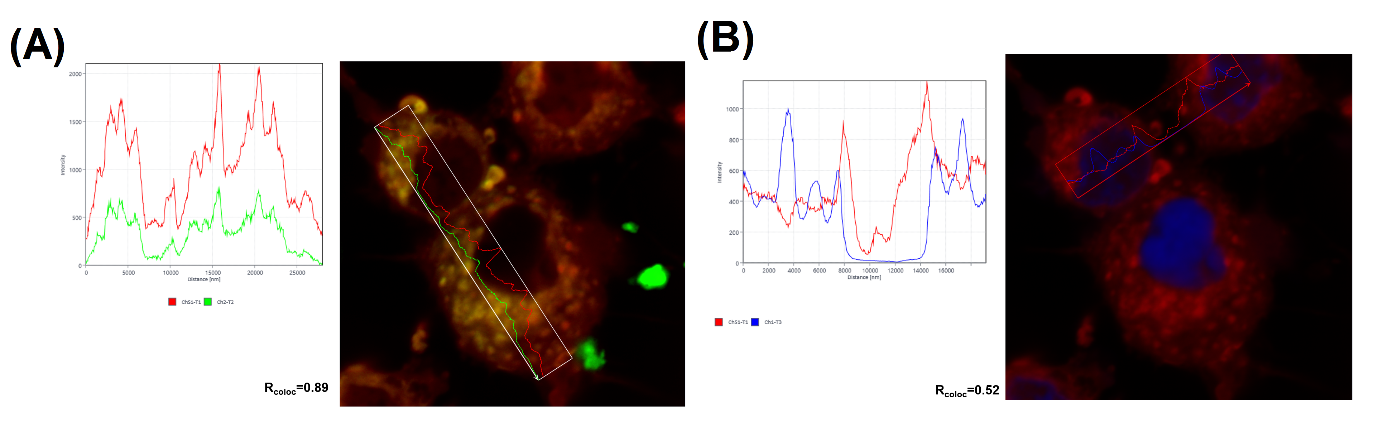


**Figure S2**. Colocalization of 1-MPSG and (A) MitoTracker and (B) Hoechst33342 in A549 cells studied by confocal microscopy and intensity profiles of 1-MPSG (red) and MitoTracker (green) or Hoechst33342 (blue) obtained using ZEN Software (Zeiss), along an arrow crossing the representative cell.


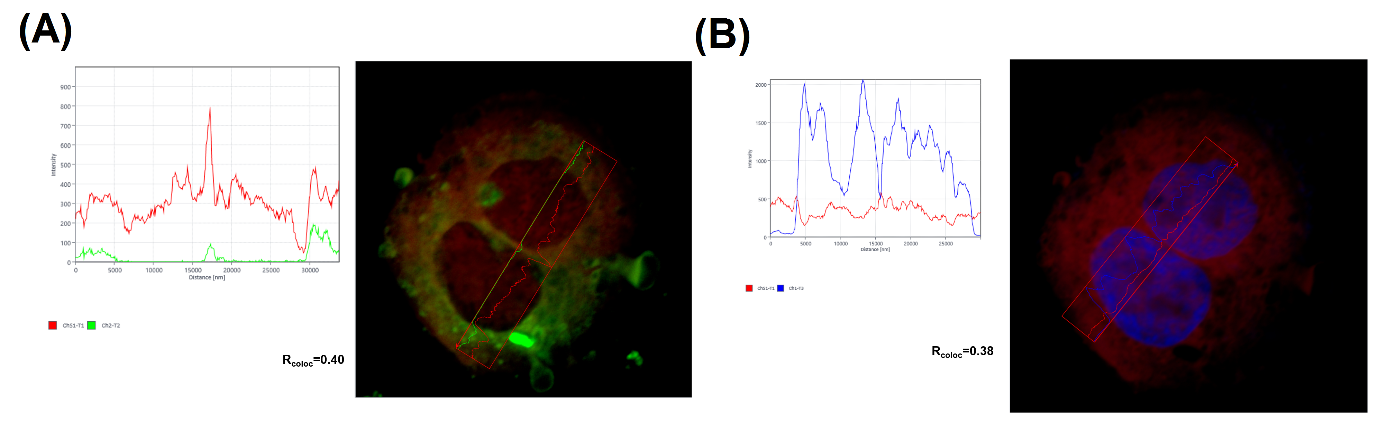


**Figure S3.**  Colocalization of 1-MPSG and (A) MitoTracker and (B) Hoechst33342 in HaCaT cells studied by confocal microscopy and intensity profiles of 1-MPSG (red) and MitoTracker (green) or Hoechst33342 (blue) obtained using ZEN Software (Zeiss), along an arrow crossing the representative cell


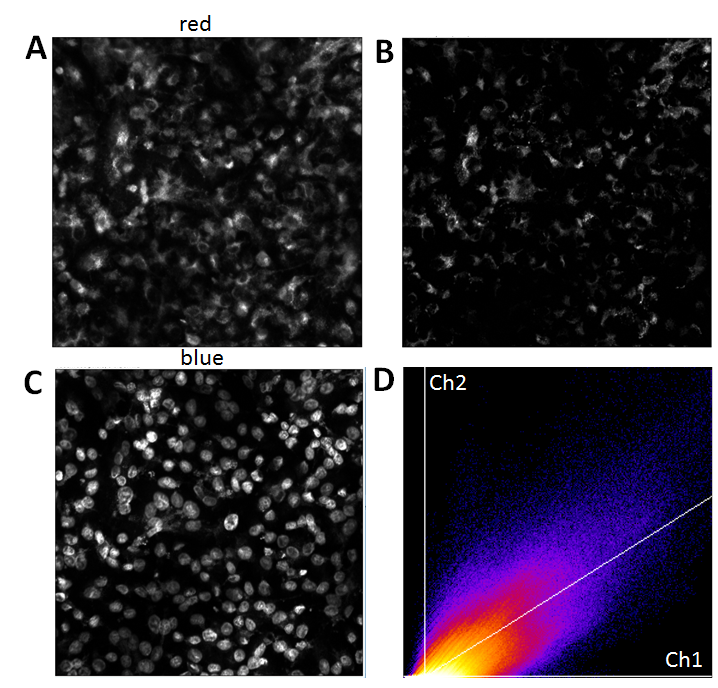


**Figure S4.** The colocalization analysis for Pearson coefficient determination prepared in Fiji ImageJ software.


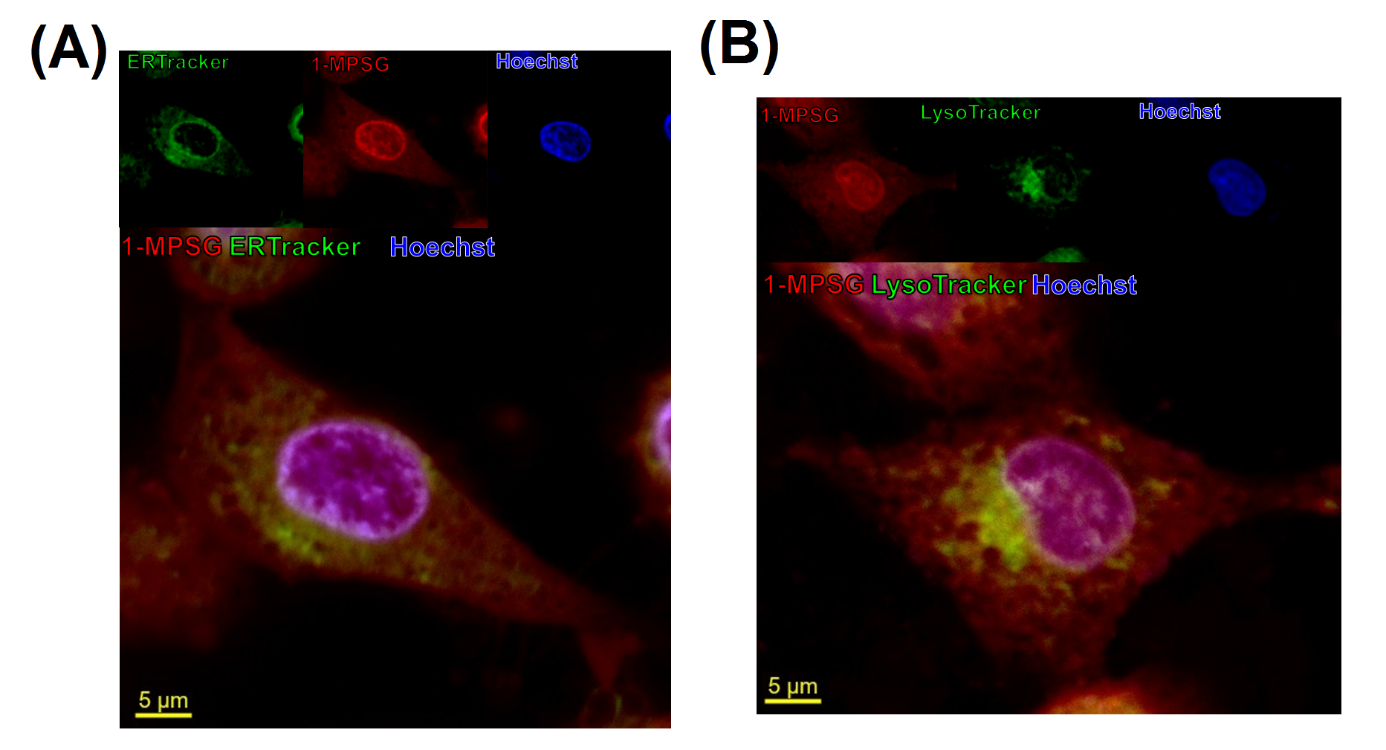


**Figure S5.** Subcellular localization of 1-MPSG in A549 cells: (A) endoplasmic reticulum was stained with ERTracker (green) and nuclei were stained with Hoechst33342 (blue); (B) lysosomes were stained with LysoTracker (green) and nuclei were stained with Hoechst33342 (blue).


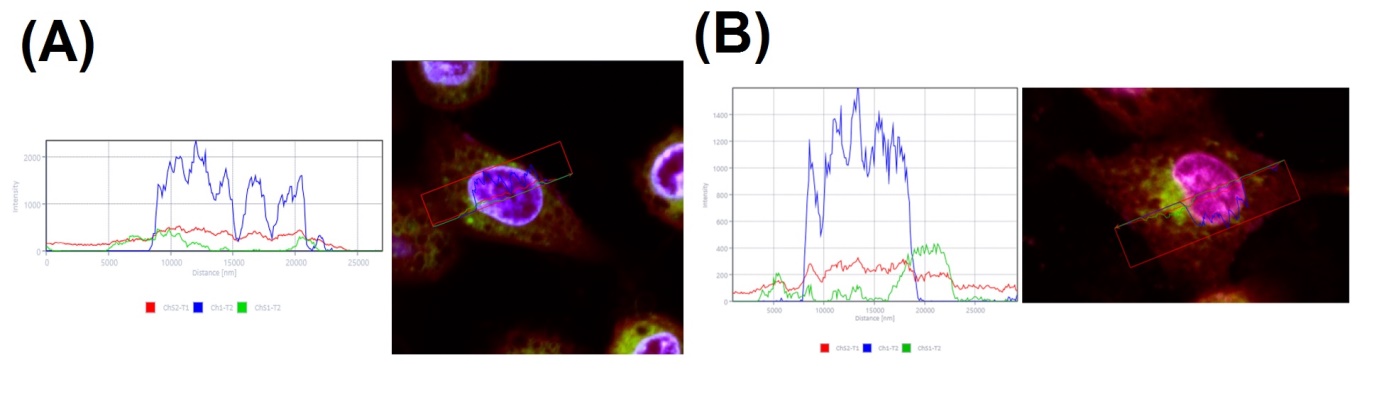


**Figure S6.** Colocalization of 1-MPSG (red) with:(A) ERTracker (green) and Hoechst33342 (blue); (B) LysoTracker (green) and Hoechst33342 in A549 cells studied by confocal microscopy with respective intensity profiles obtained using ZEN Software (Zeiss), along an arrow crossing the representative cell.


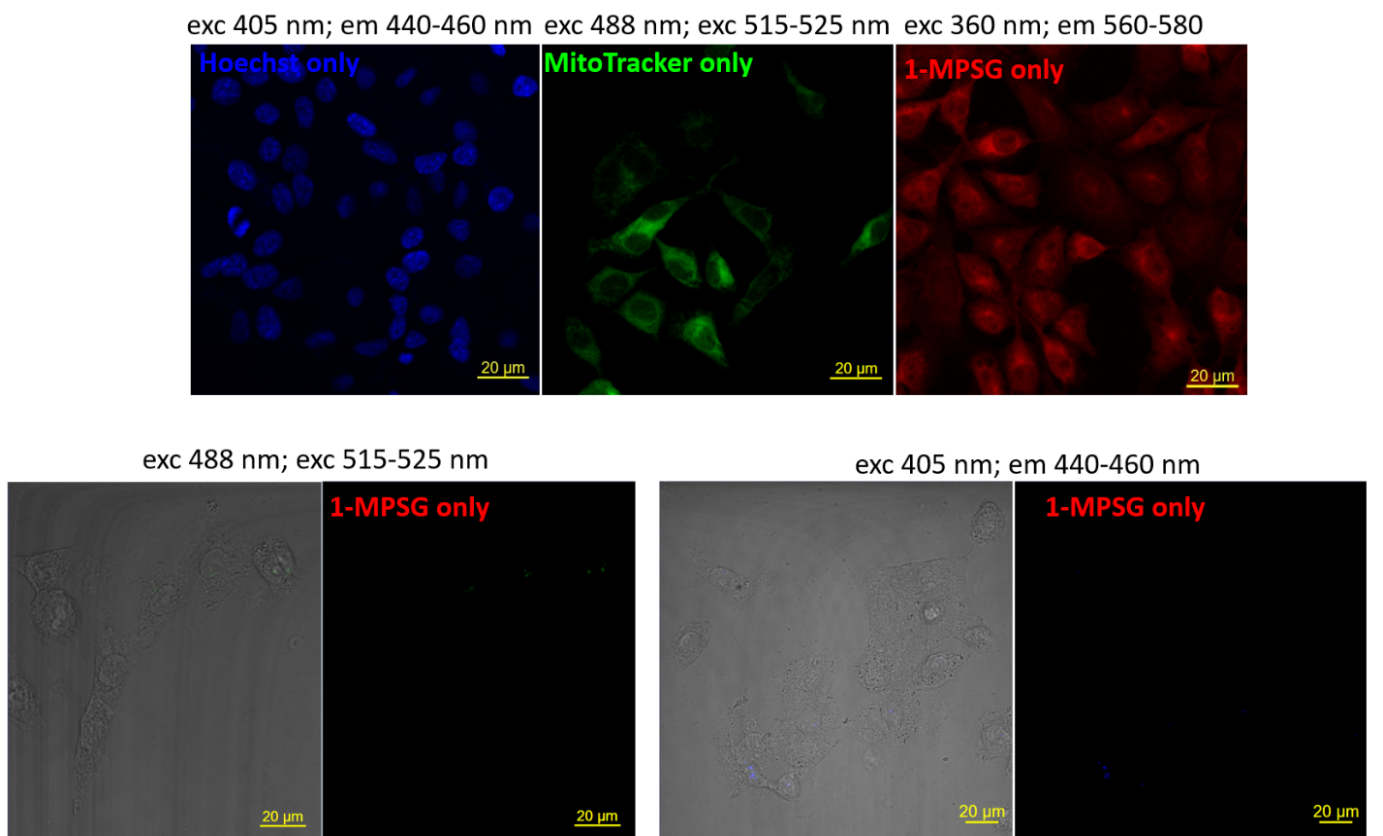


**Figure S7.** Single stained controls of Hoechst 33342, MitoTracker Green, and **1-MPSG**.


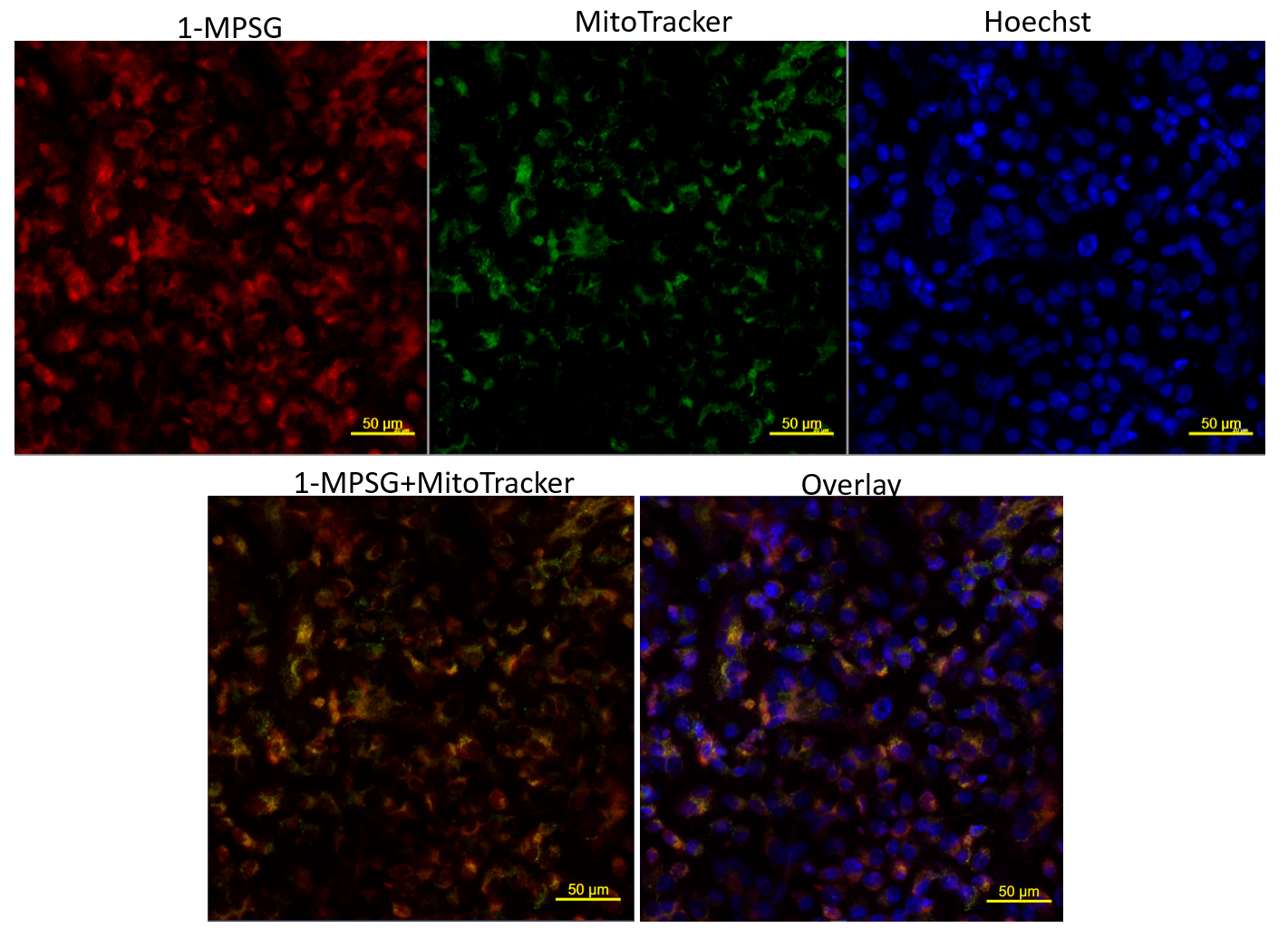


**Figure S8.** Single stained controls and colocalization images of **1-MPSG** with MitoTracker Green in (A) A549 cells. The cells were treated with 1 µM **1-MPSG** for 2h and then co-incubated with 200 nM MitoTracker Green for 1h and counterstained with Hoechst 33342.


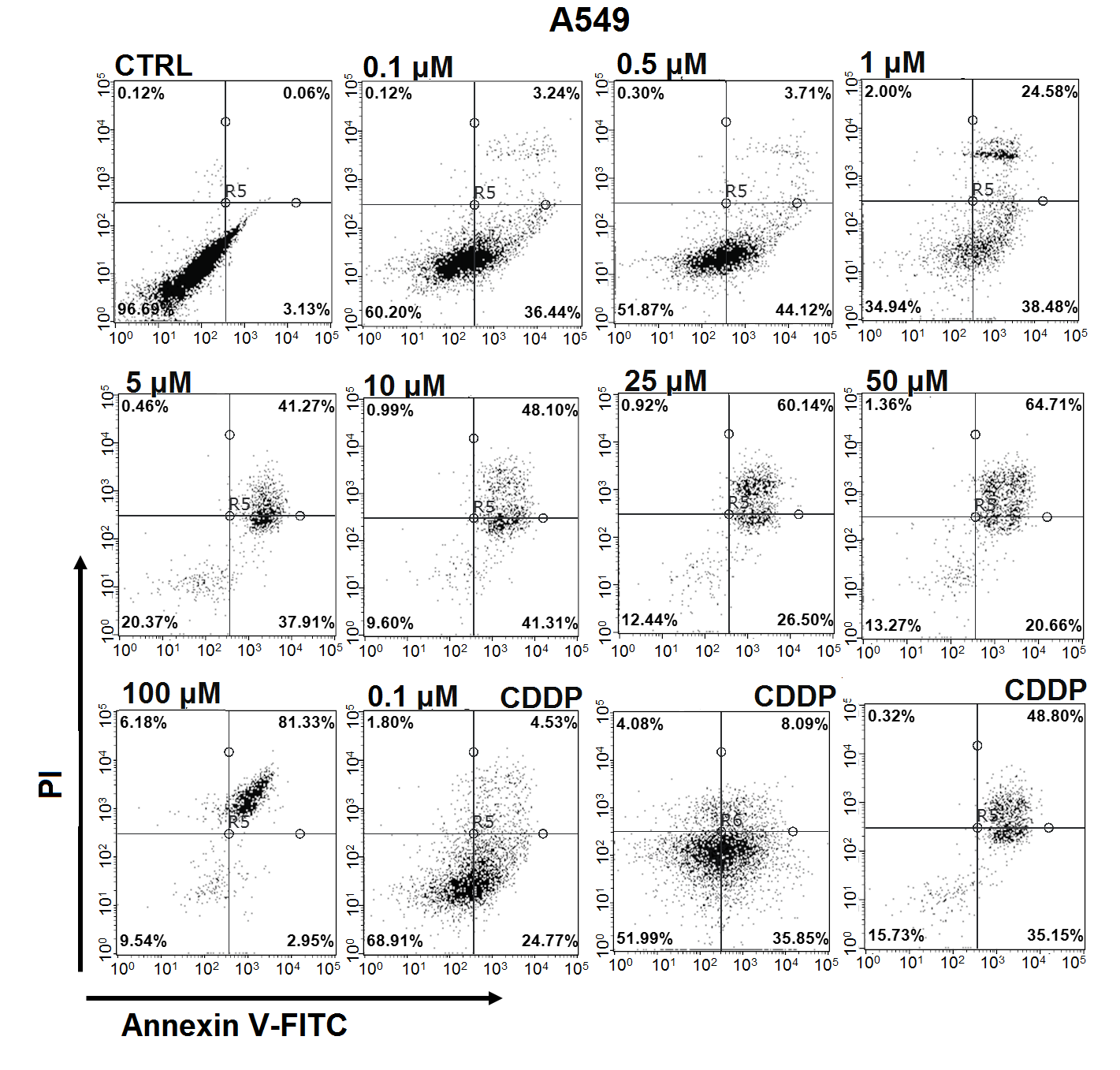


**Figure S9.** Representative dotplots obtained by flow cytometry showing determination of A549 cell death modes induced by **1-MPSG** and cisplatin (CDDP): the Annexin V-FITC/propidium iodide (PI) double staining assay (Annexin V-FITC - green fluorescence, PI - red fluorescence) was used to detect phosphatidylserine externalization in apoptosis and analyse the membrane integrity, respectively.


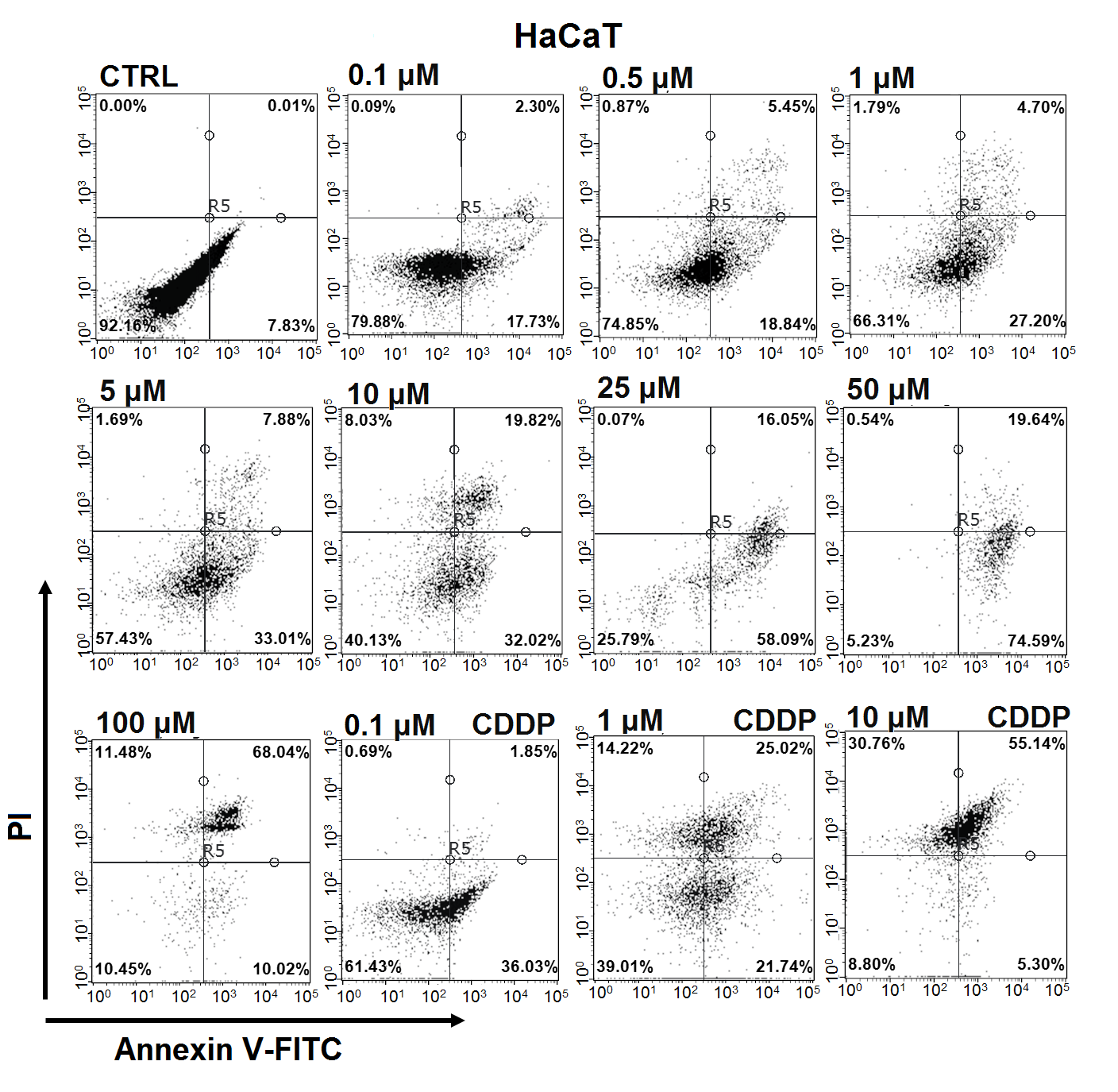


**Figure S10.** Representative dotplots obtained by flow cytometry showing determination of HaCaT cell death modes induced by **1-MPSG** and cisplatin (CDDP): the Annexin V-FITC/propidium iodide (PI) double staining assay (Annexin V-FITC - green fluorescence, PI - red fluorescence) was used to detect phosphatidylserine externalization in apoptosis and analyse the membrane integrity, respectively.


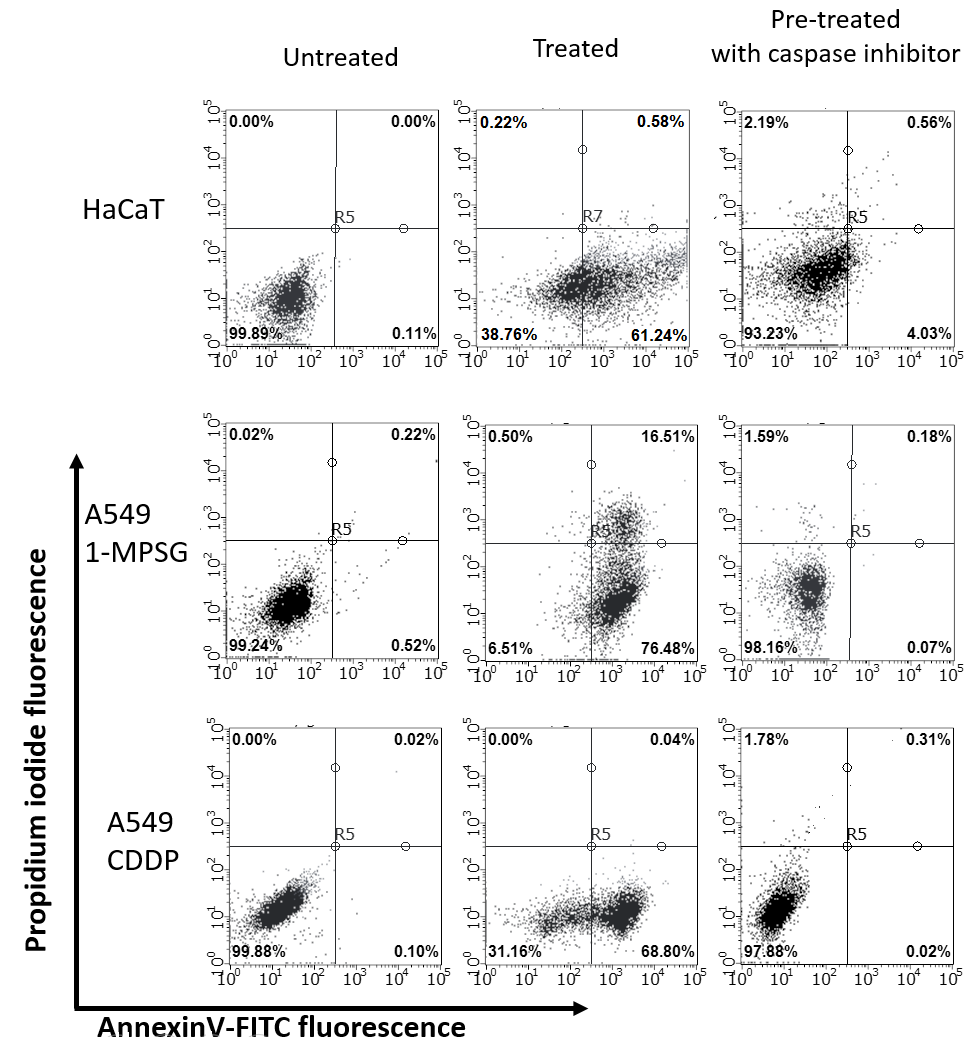


**Figure S11.** The effect of caspase inhibitor (Z-VAD-FMK) on the HaCaT cells treated with 1μM 1-MPSG, A549 cells treated with 1μM 1-MPSG and A549 cells treated with 1μM CDDP; Data are expressed as mean ± SEM.


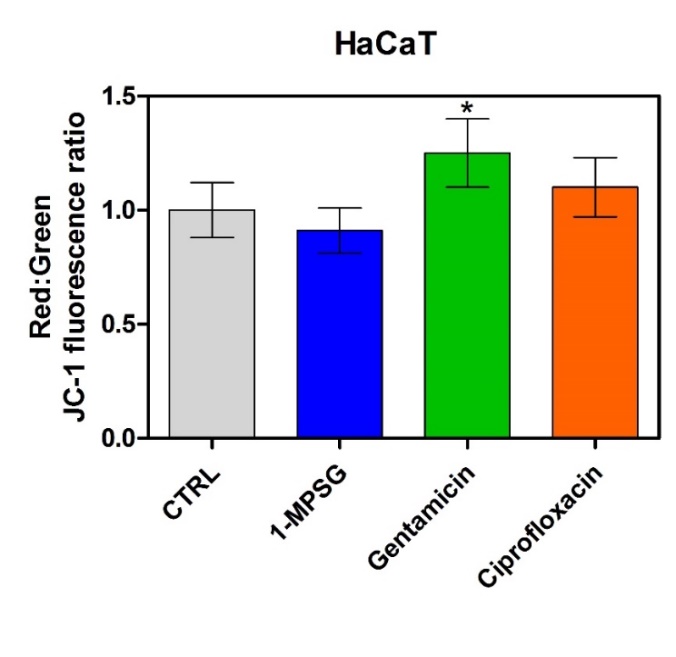


**Figure S12.** Influence of **1-MPSG** complexes (at IC_50_) on the intensity of JC-10 fluorescence in treated HaCaT cells. Alteration in MMP is given as an emission ratio 570 nm/530 nm. (control – untreated cells, ciprofloxacin – a negative control, gentamicin – a positive control); Data are present as mean±SEM; * P<0.05 compared to untreated control CTRL)


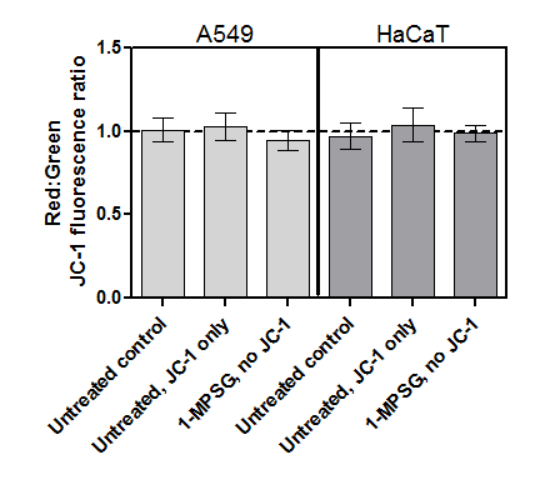


**Figure S13.** Controls for the experiment indicating the influence of 1-MPSG complexes (at IC_50_) on the intensity of JC-10 fluorescence in treated A549 and HaCaT cells. Alteration in MMP is given as an emission ratio 570 nm/530 nm. (control – untreated cells, ciprofloxacin – a negative control, gentamicin – a positive control); Data are present as mean±SEM; * P<0.05 compared to untreated control CTRL)


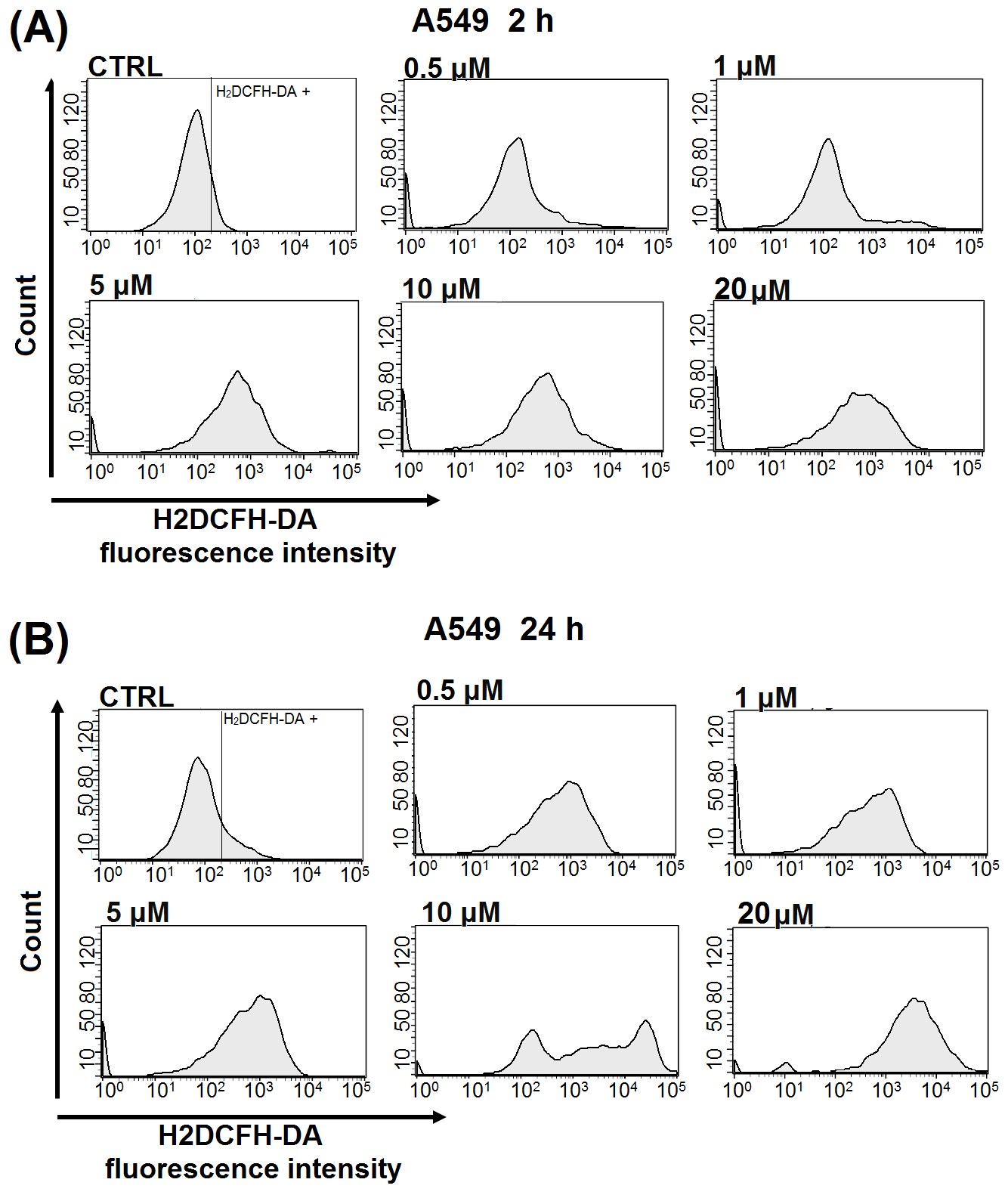


**Figure S14.** Flow cytometry analysis of ROS formation induced **by 1-MPSG** using the H2DCFH-DA probe in A549 cells after (A) 2h and (B) 24 h incubation with **1-MPSG** at various concentrations.


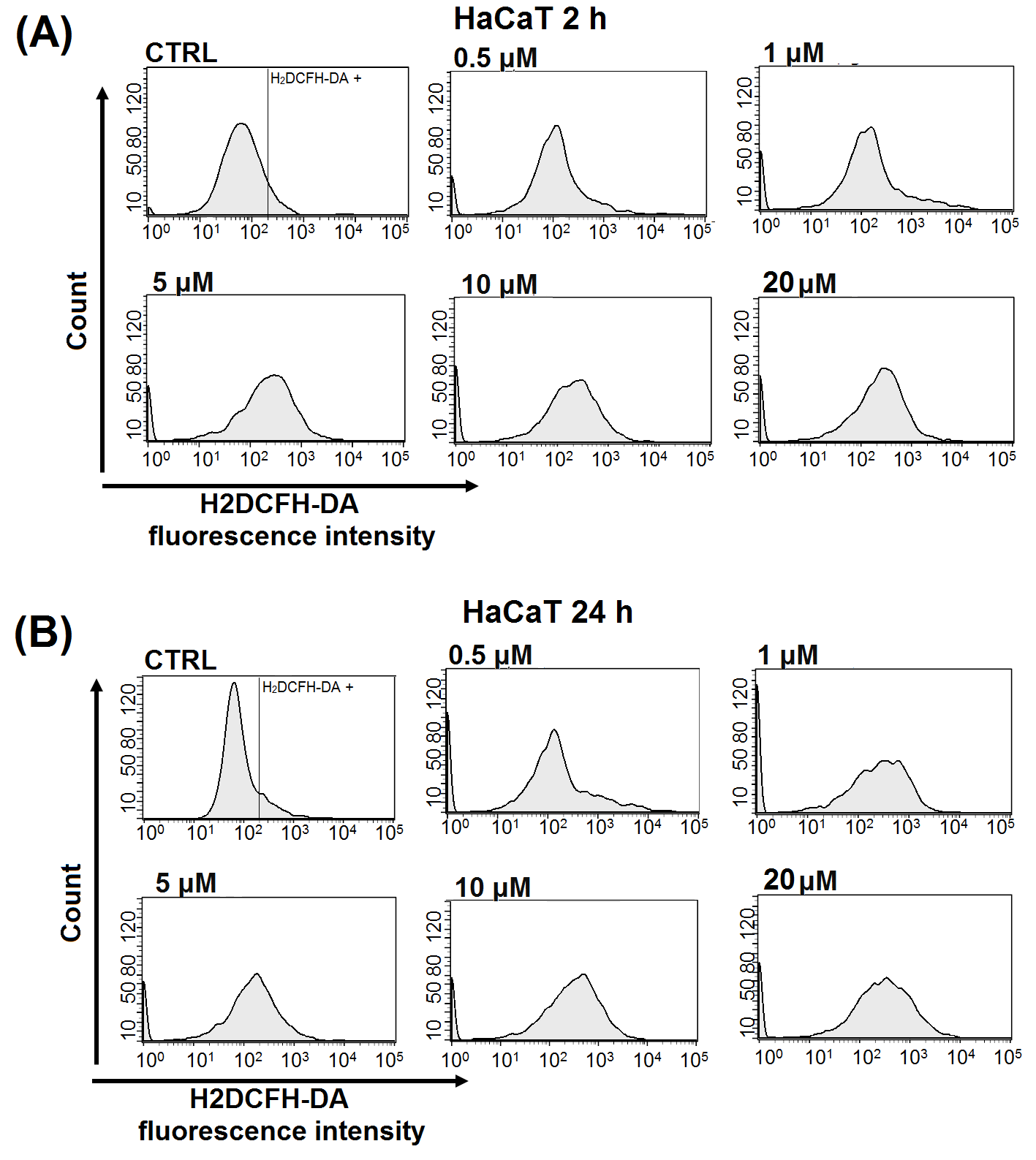


**Figure S15.** Flow cytometry analysis of ROS formation using the H2DCFH-DA probe in HaCaT cells after (A) 2h and (B) 24 h incubation with **1-MPSG** at various concentrations.


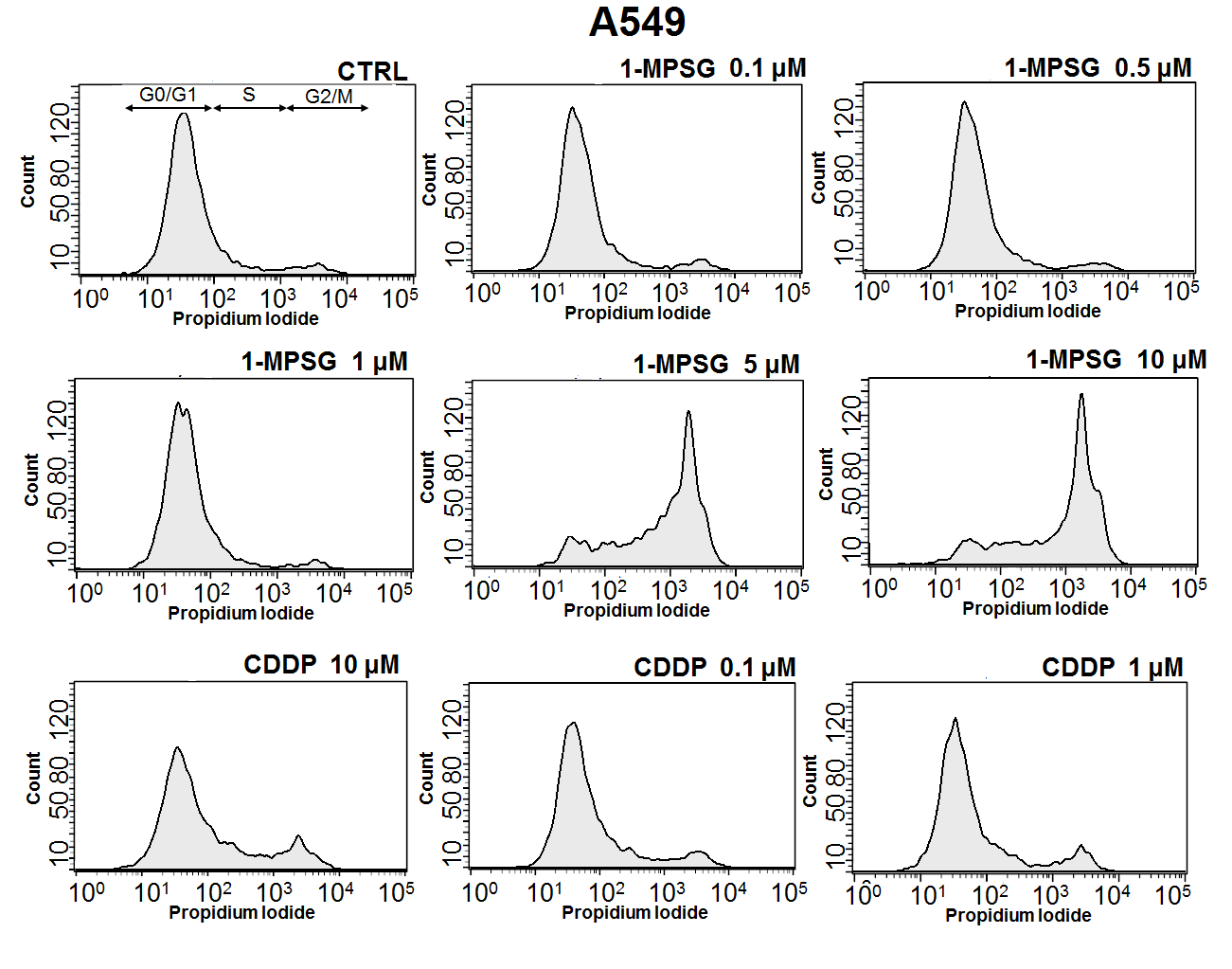


**Figure S16.** Representative histograms of A549 stained with Propidium Iodide (PI) showing DNA content distribution after the 24 h treatment with **1-MPSG** and cisplatin (CDDP) at various concentrations. G0/G1 and G2/M phase histogram peaks are separated by the S-phase distribution. Prior to experiments, cells were synchronized.


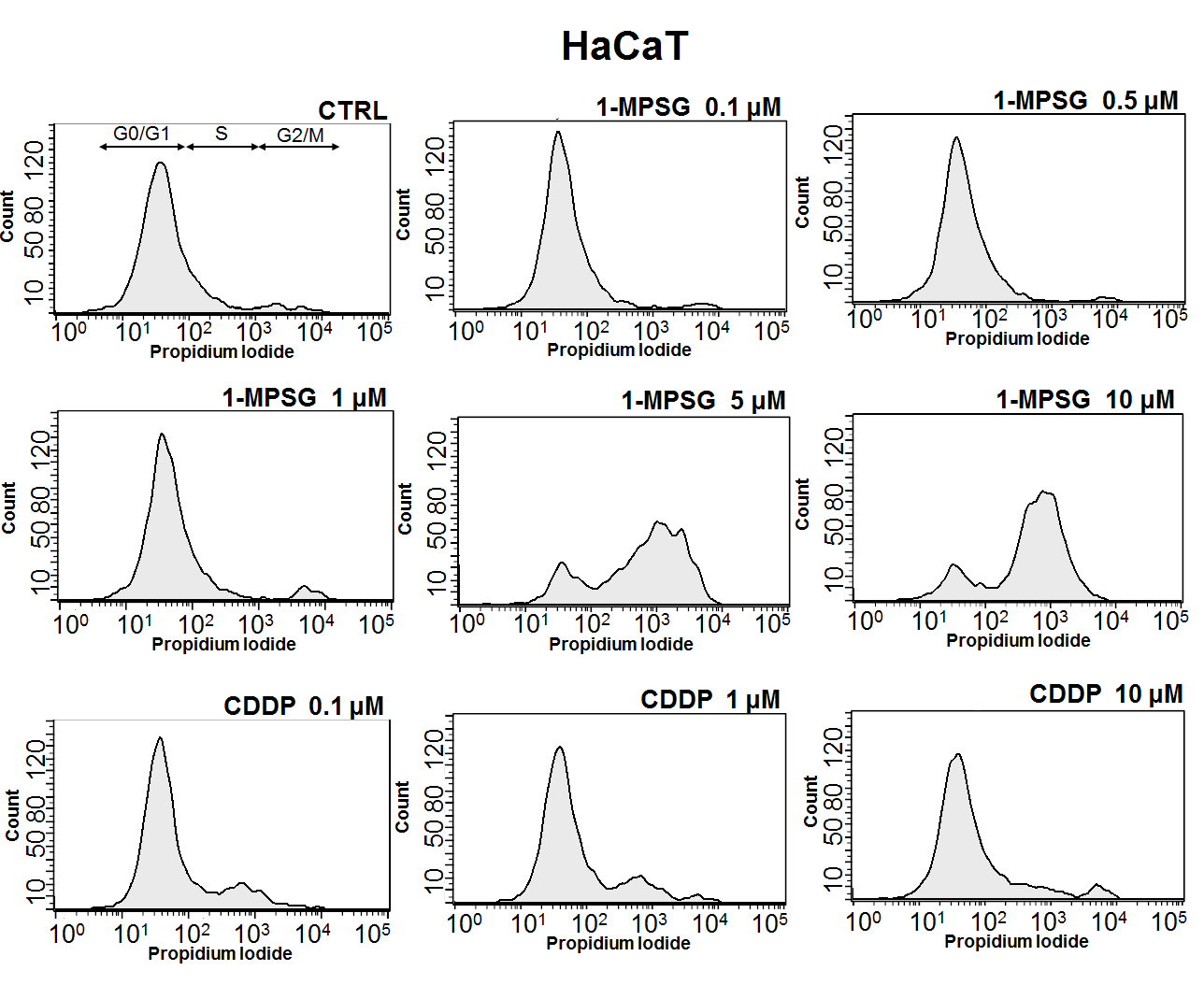


**Figure S17.** Representative histograms of HaCaT stained with Propidium Iodide (PI) showing DNA content distribution after the 24 h treatment with **1-MPSG** and cisplatin (CDDP) at various concentrations. G0/G1 and G2/M phase histogram peaks are separated by the S-phase distribution. Prior to experiments, cells were synchronized.


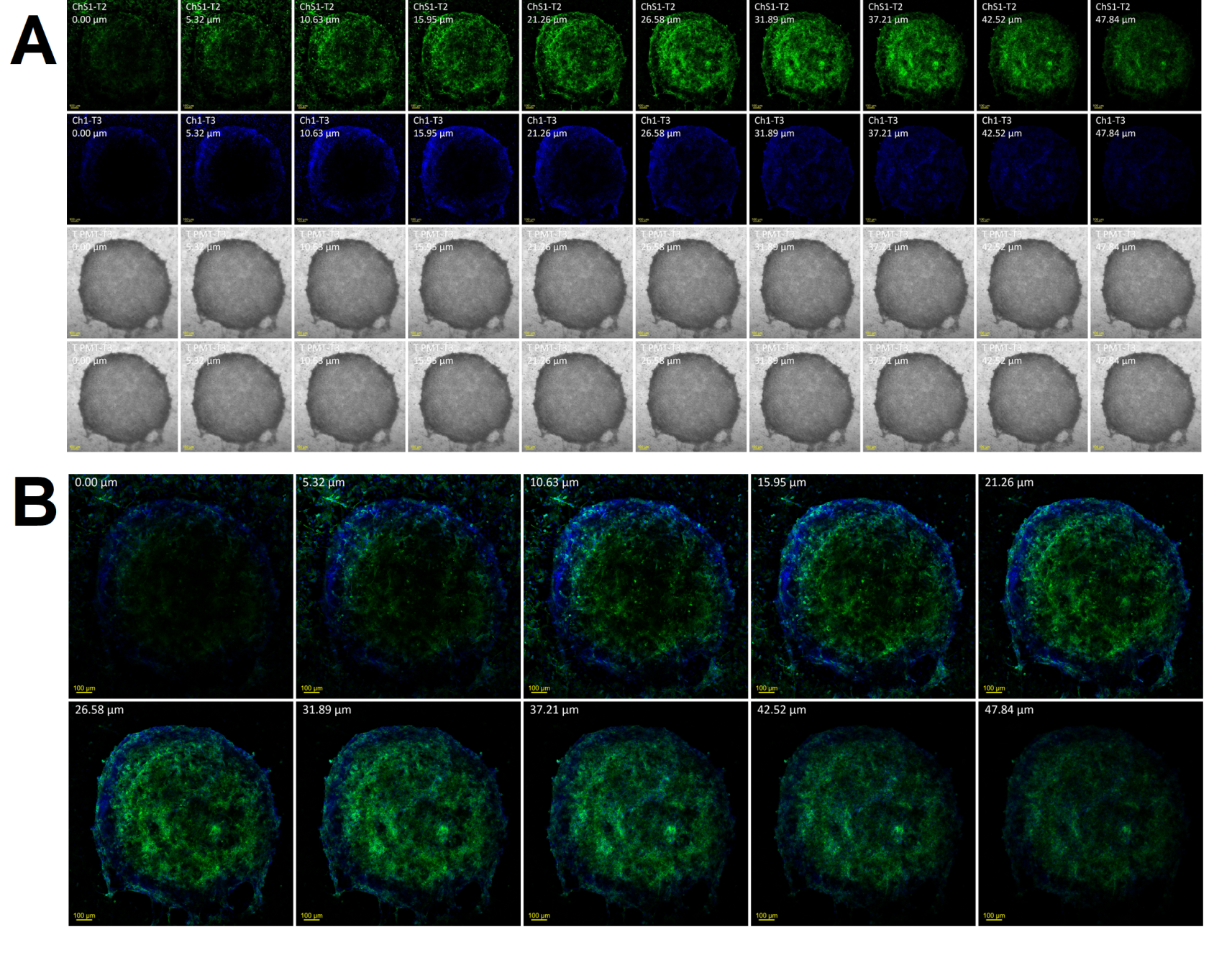


**Figure S18.** A549 spheroids characterization – z-stack images obtained for representative spheroid stained with Hoechst33342 (blue) and Phalloidin-FITC (green).


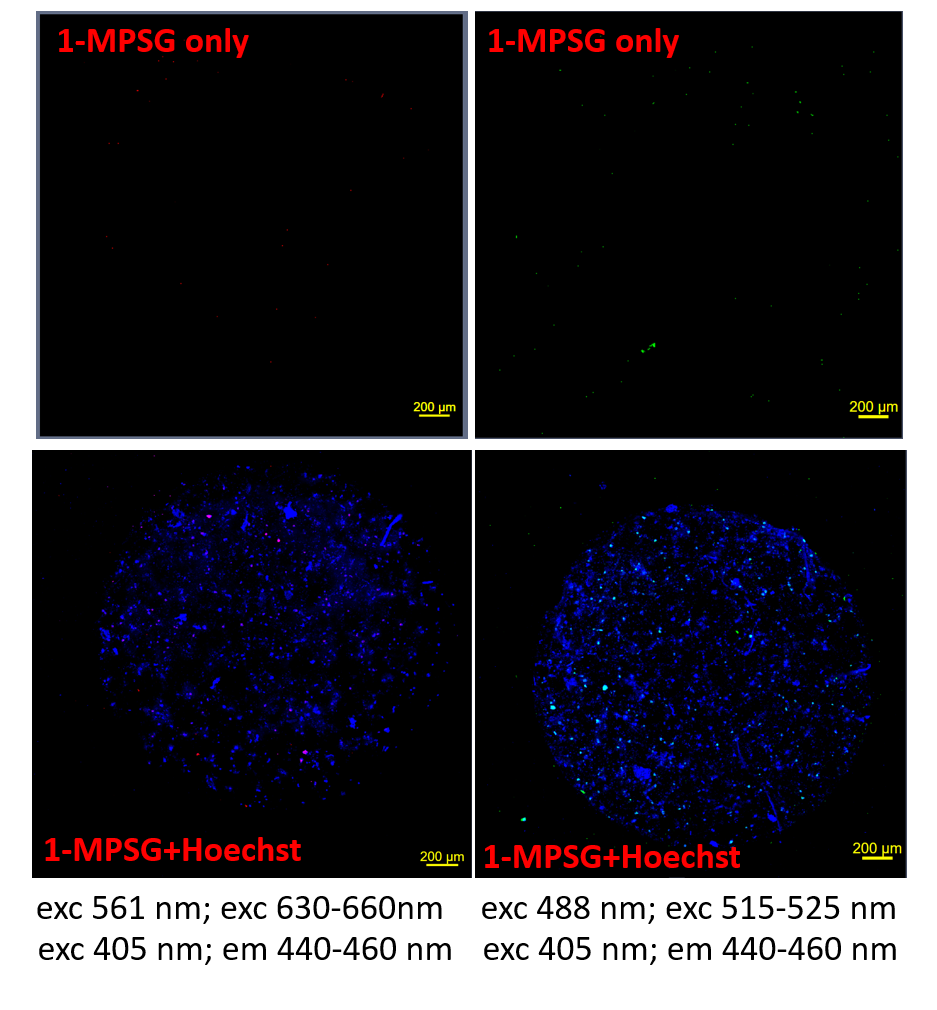


**Figure S19**. **1-MPSG** fluorescence control for A549 spheroid staining.
